# Correction: Development of an open-hardware semen homogenizer and application to serotonin effects on sperm motility

**DOI:** 10.1371/journal.pone.0345800

**Published:** 2026-03-24

**Authors:** The PLOS One Staff

S2 Video is a duplicate of S1 Video. Please view the correct S2 Video below.

The publisher apologizes for the error.

## Supporting information

S2 Video CASHo in operation. (MP4)
