# Supplementary material for: Development of an open-hardware semen homogenizer and application to serotonin effects on sperm motility
Source: PLoS One. 2025 Dec 5;20(12):e0338399. doi: 10.1371/journal.pone.0338399 (PMC12680138; doi:10.1371/journal.pone.0338399)
Supplement: S1 File — (DOCX) [file pone.0338399.s001.docx]

## Description of CASHo

The CASHo was designed to provide homogeneous mixing and reduce operator variability during the handling of cell suspensions. The system features a fully customizable structure, encompassing both the aluminum framework and the firmware, allowing users to adjust the device according to specific laboratory requirements. Its tube holder was conceived to tubes of different capacities, ensuring versatility across experimental setups. In addition, the rotation speed, angle, and number of cycles can be configured through the firmware, offering precise control over the mixing process. The design is also scalable, permitting expansion of the tube holder to process a higher number of samples when needed.

Although developed primarily for semen homogenization, the CASHo was intended to enable objective mixing of other types of cellular suspensions where reproducible mechanical agitation is desirable.

CASHo consists of an extruded aluminum base and a set of 3D-printed components that support a stepper motor and custom-made parts designed to hold a 50 mL tube. The electronic control board is an Arduino UNO, which receives instructions via Arduino App ([https://www.arduino.cc](https://www.arduino.cc/)) through a computer serial monitor. The tube holder performs an inverted pendular motion at a 180-degree angle, a movement selected because it replicates the hand motion during manual homogenization (see the video Hand_homogenization.mp4 in the Videos section of

<https://doi.org/10.17605/OSF.IO/ESFWG>).

Several components of CASHo were designed for 3D printing using OpenSCAD (https://openscad.org) (Table 1). The design files (STL), code source (scad), and supporting information are in the STL section at https://doi.org/10.17605/OSF.IO/ESFWG; whereas details on design files, components and mounting details are in the file CASHo _Supplementary_Details.pdf in the Supplementary section at https://doi.org/10.17605/OSF.IO/ESFWG.

## Design files descriptions


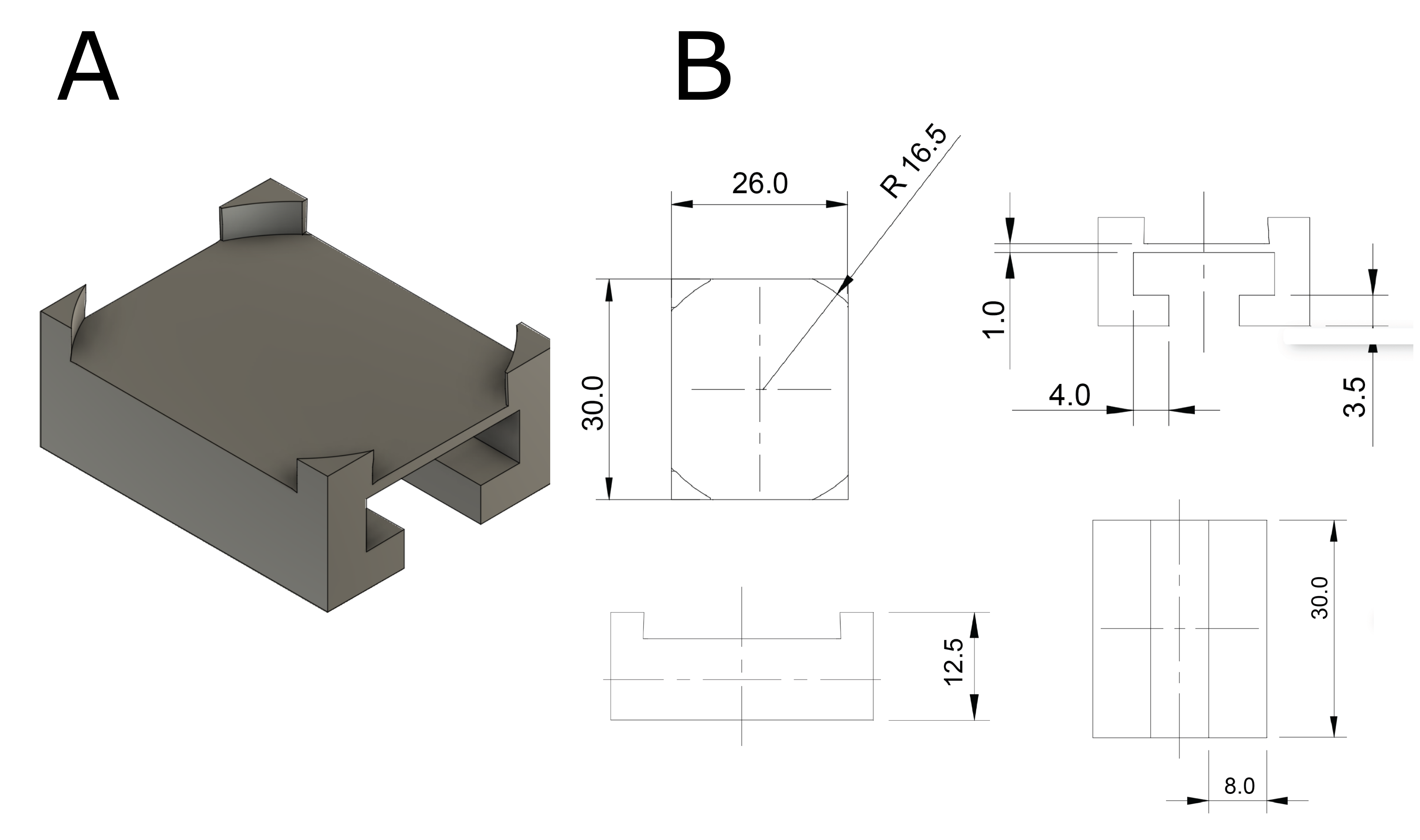


**Fig 1. Junction piece dimensions.** Junction_piece.stl is a part where 1) the base of the Tube_holder is attached, and 2) it is coupled to the Rod_adapter via a T-slot sliding assembly (Fig 1).


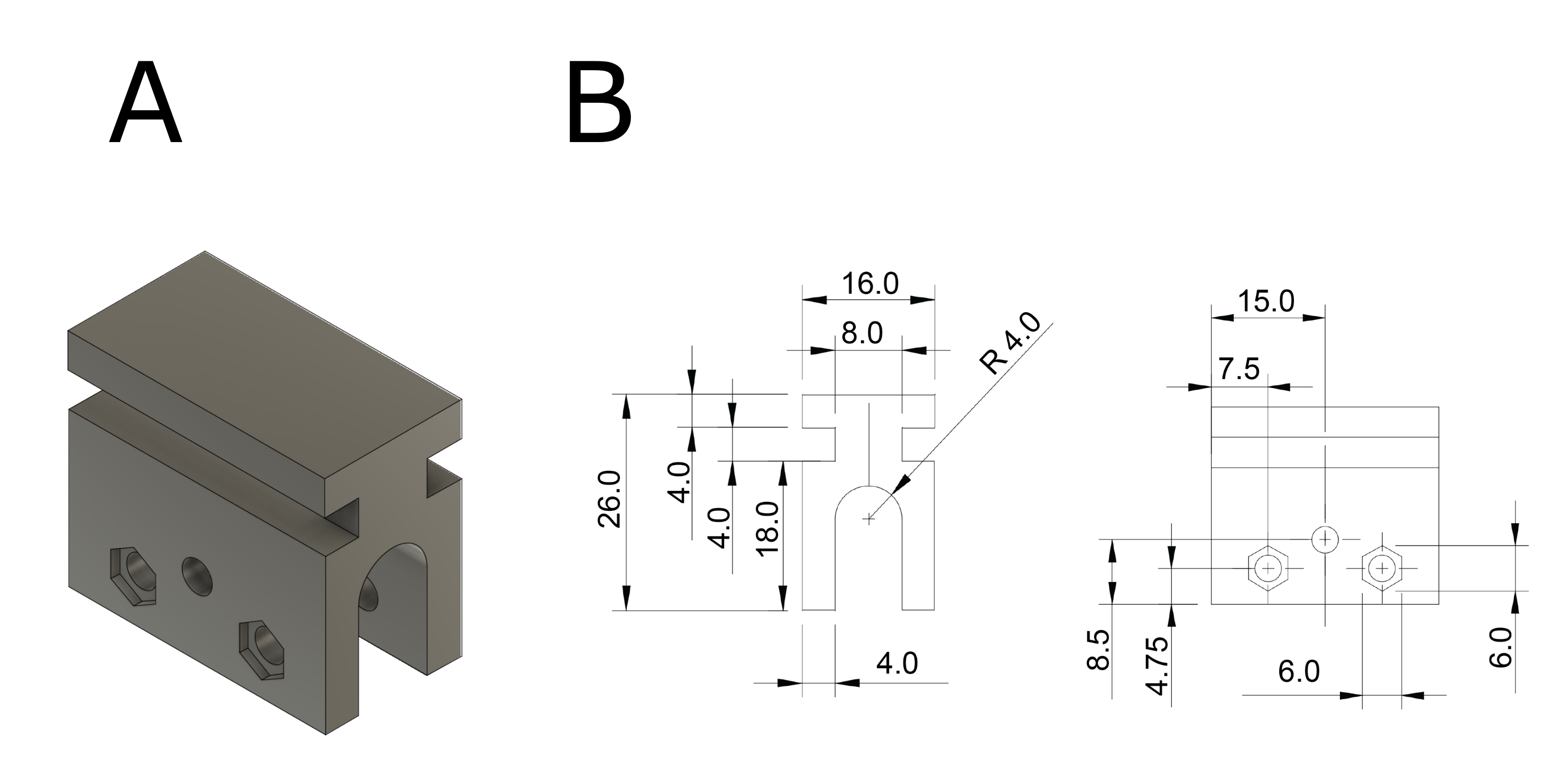


**Fig 2. Rod adapter dimensions.** Rod_adapter.stl is a component designed to fit an 8 mm rod. It features openings for 3 mm screws and integrated slots for securing nuts. Its primary function is to hold the Junction_piece and Tube_holder firmly in place (Fig. 2).


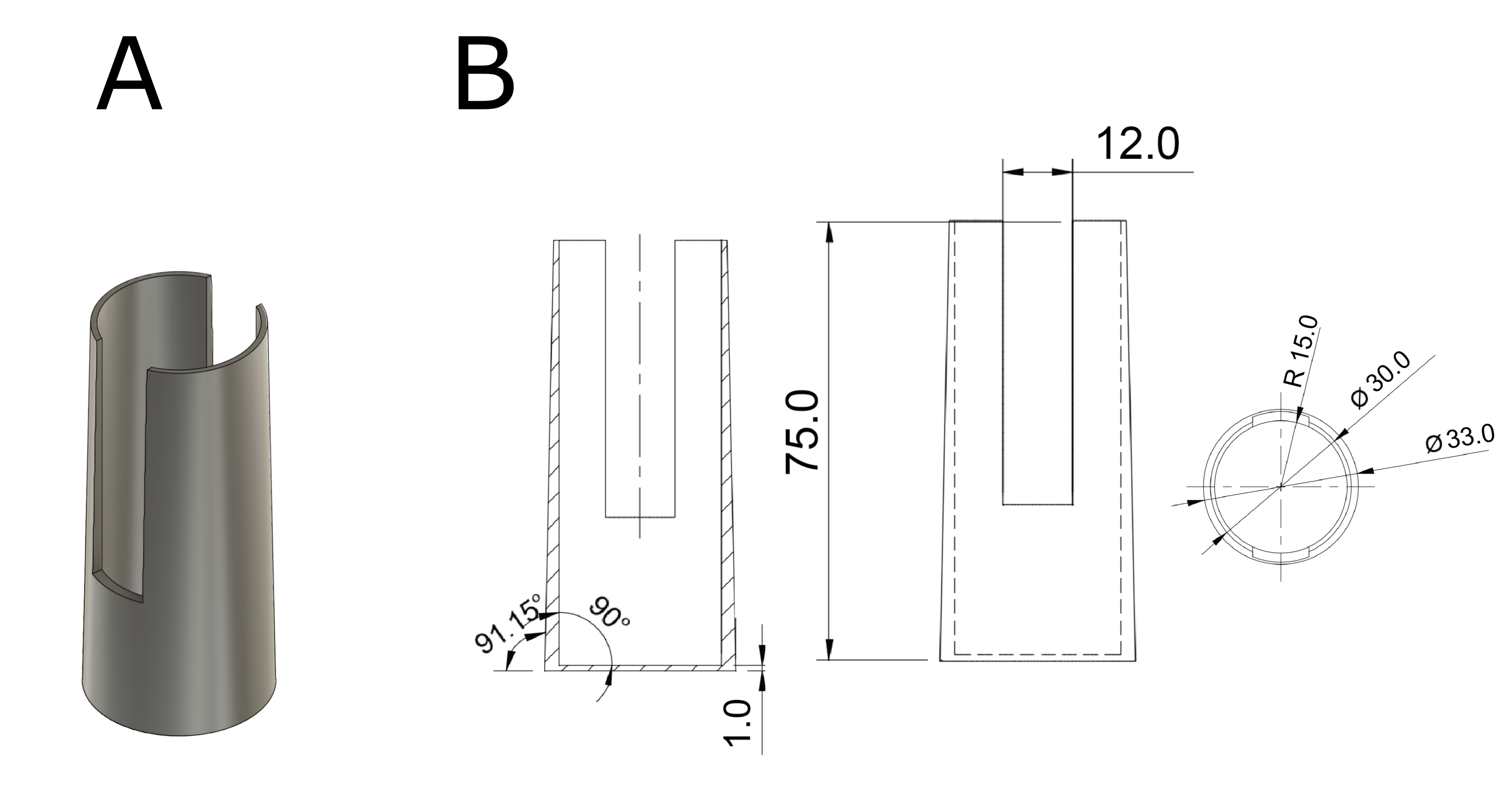


**Fig 3. Tube holder dimensions.** Tube_holder.stl is a sleeve with a height of 7.5 cm and a flat base, designed to hold a 50 mL tube. The sleeve has two 1 cm openings positioned 180° apart, allowing observation of the tube's contents (Fig 3).


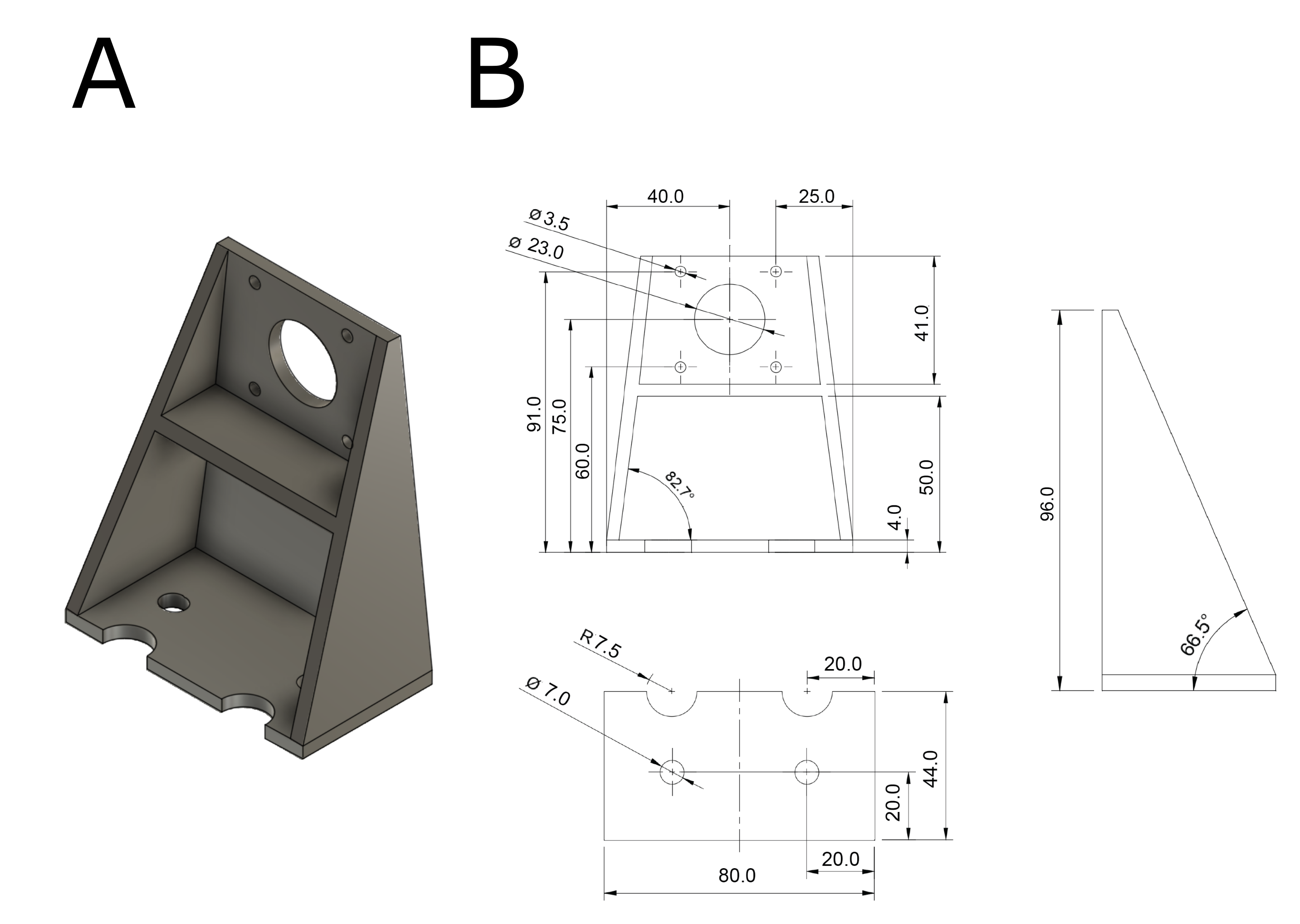


**Fig 4. Nema support dimensions.** Nema_support.stl is the part that holds the Nema17 motor 5 cm above the horizontal extruded aluminum piece. It has 4 holes for securing the motor with 3 mm screws, and 2 holes at the base for fastening the part to the horizontal extruded aluminum piece (Fig 4).


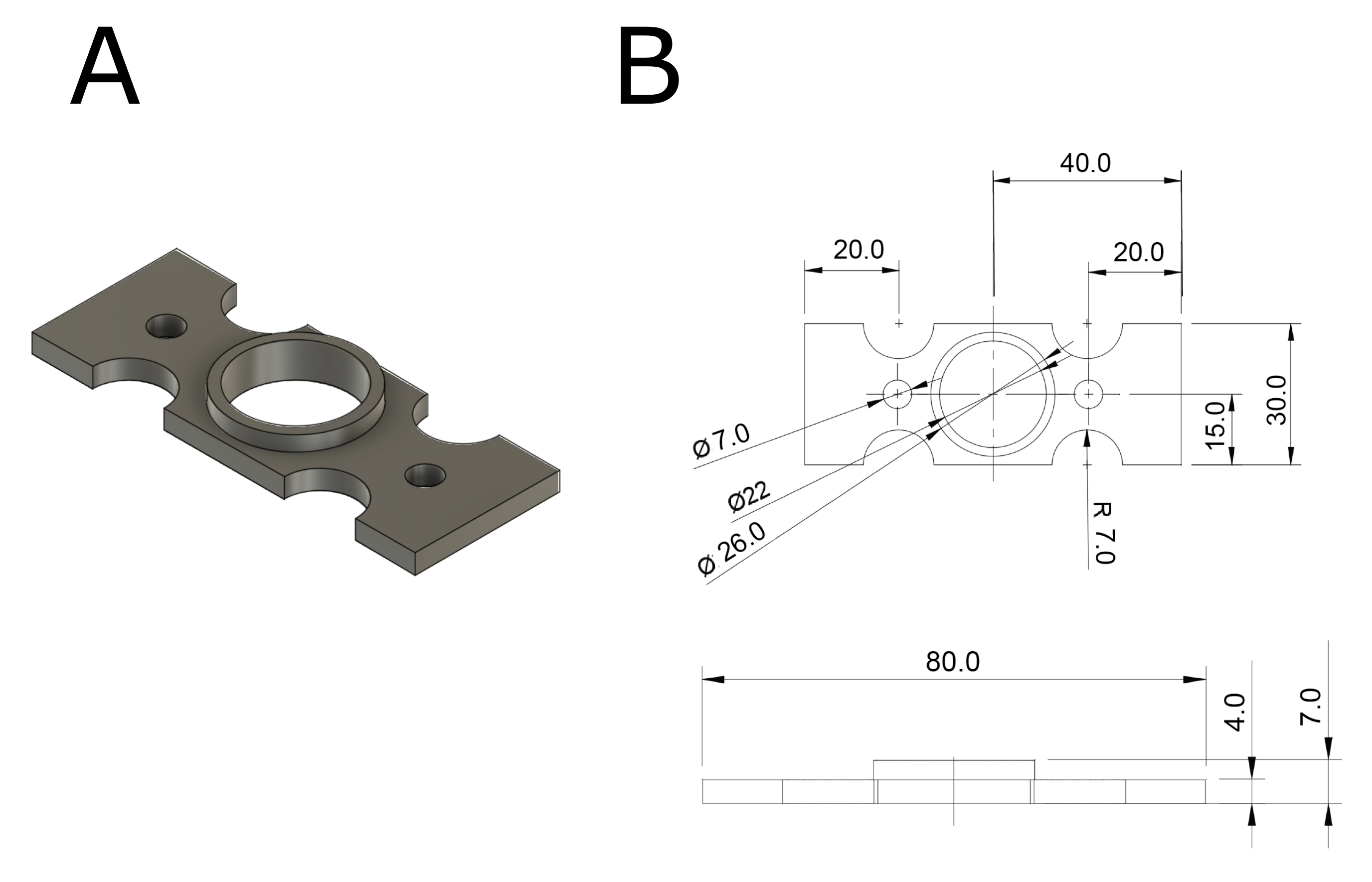


**Fig 5. Rod support dimensions.** Rod_support.stl is a component with two holes that allow it to be mounted onto the vertical extruded aluminum piece. It houses a 608RS bearing at its center. An 8 mm rod is inserted through the bearing, with its other end connected to the motor via a flexible aluminum coupling (Fig 5).

## Build instructions

The horizontal extruded aluminum piece was drilled (1/8 inch), and threading was done using a tap (1/8 inch). In Mexico, this work is typically performed by the aluminum supplier.

To construct the frame of CASHo we used two pieces of 40 mm x 80 mm metric series rectangular T-slot profile with six open T-slots, two on each 80 mm face and one on each 40 mm face; one piece of 350 mm y other of 150 mm. The larger piece of aluminum is intended to serve as the horizontal base, while the shorter piece is designed to be joined at a 90° angle to the larger piece. Both pieces are secured in place with the aid of two end fasteners and two 30 mm x 40 mm nuts. We used commercial end cap with push-in fastener closed off profile ends (Fig 6).


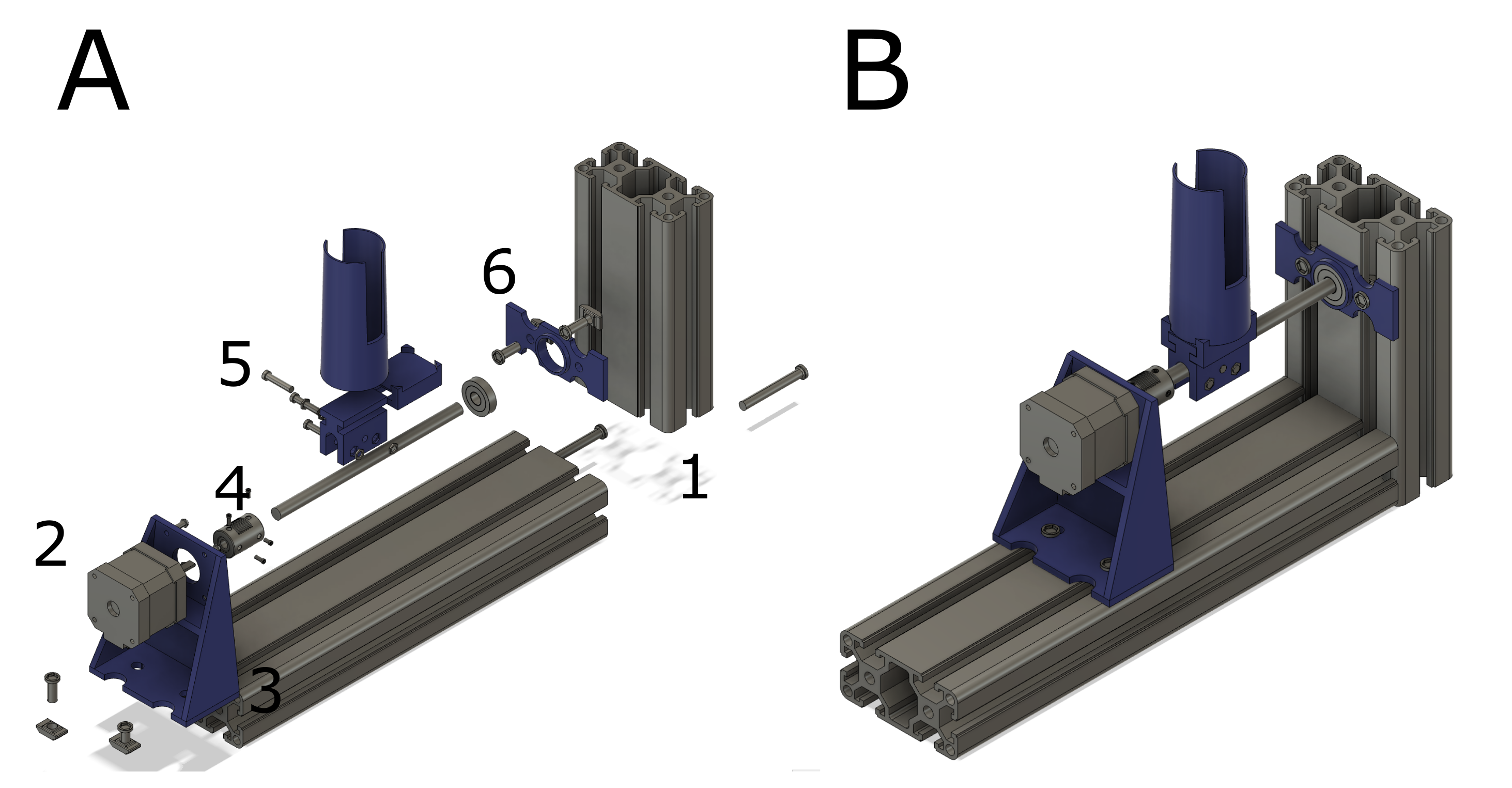


**Fig 6. Exploded and assembled view of CASHo.** Numbers in (A) indicate the steps of assembly: 1) Attach and securely assemble the horizontal and vertical pieces of extruded aluminum using bolts and secure fasteners. 2) Attach and screw the Nema motor to the Nema_support piece. 3) Smoothly screw the Nema_support to the horizontal piece of extruded aluminum. 4) Screw the flexible coupling to the shaft of the Nema motor and to the rod. 5) Screw the Rod_adapter to the rod. 6) Push the 608RS bearing into the hole of the Rod_support, and screw the Rod_support to the vertical piece of extruded aluminum. (B) shows CASHo assembled without electronics.The stepper motor nema17 was screwed to the Nema_support with with four M5x3 mm bolts. Then Nema_support was lightly secured with two M4 T-nuts and two M4x5 mm bolts to the horizontal aluminum base (displacement of the Nema_support must be guaranteed) (Fig 6). A 608RS bearing was pushed into the central hole of the rod_adapter (Fig 6).

The rod_support was secured with two M4 T-nuts and two M4x5 mm bolts to the vertical aluminum piece, at a distance of 80 mm from the upper ending of the horizontal aluminum base (Fig 6). A flexible aluminum coupling 5 mm x 5 mm was secured to the shaft of the motor and one end of the steel rod (Fig 6). The other end of the steel rod must be to put into the bearing of the rod_support (Fig 6). At this point, the screws securing the Nema_support, to the aluminum base, must be firmly secured (Fig 6).

### Electronic components

One end of the wire connectors was attached to the stepper motor, while the other end was connected to the motor driver. Building the electronics consists in correctly wiring the Arduino UNO board to the TB6600 driver and the Nema17 stepper motor to TB6600 driver. The pin wiring is described in the file Homogenizador_v6.ino (<https://doi.org/10.17605/OSF.IO/ESFWG>) and is graphically illustrated in Fig. 7.


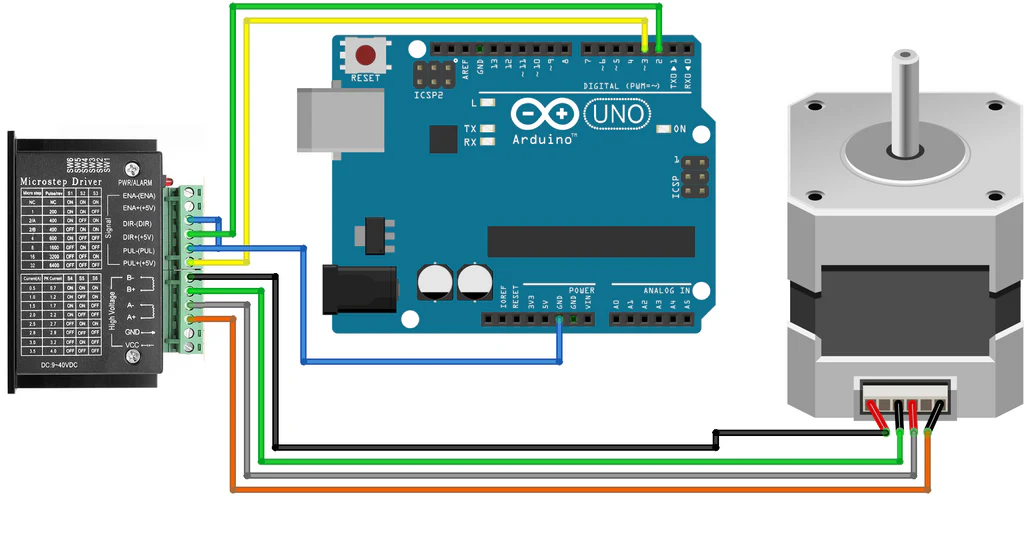


**Fig 7. Wiring the Arduino UNO board to the TB6600 driver.**

### Power delivery

For the testing assembly, power was initially supplied to the TB6600 driver using the 12V output from a LC-B350ATX power supply, which was recycled from an old computer (Fig 8). For the final assembly, a generic power supply (Part #1 in Table 2) was used (See the video CASHo_homogenizing_process.mp4 at <https://doi.org/10.17605/OSF.IO/ESFW>) .


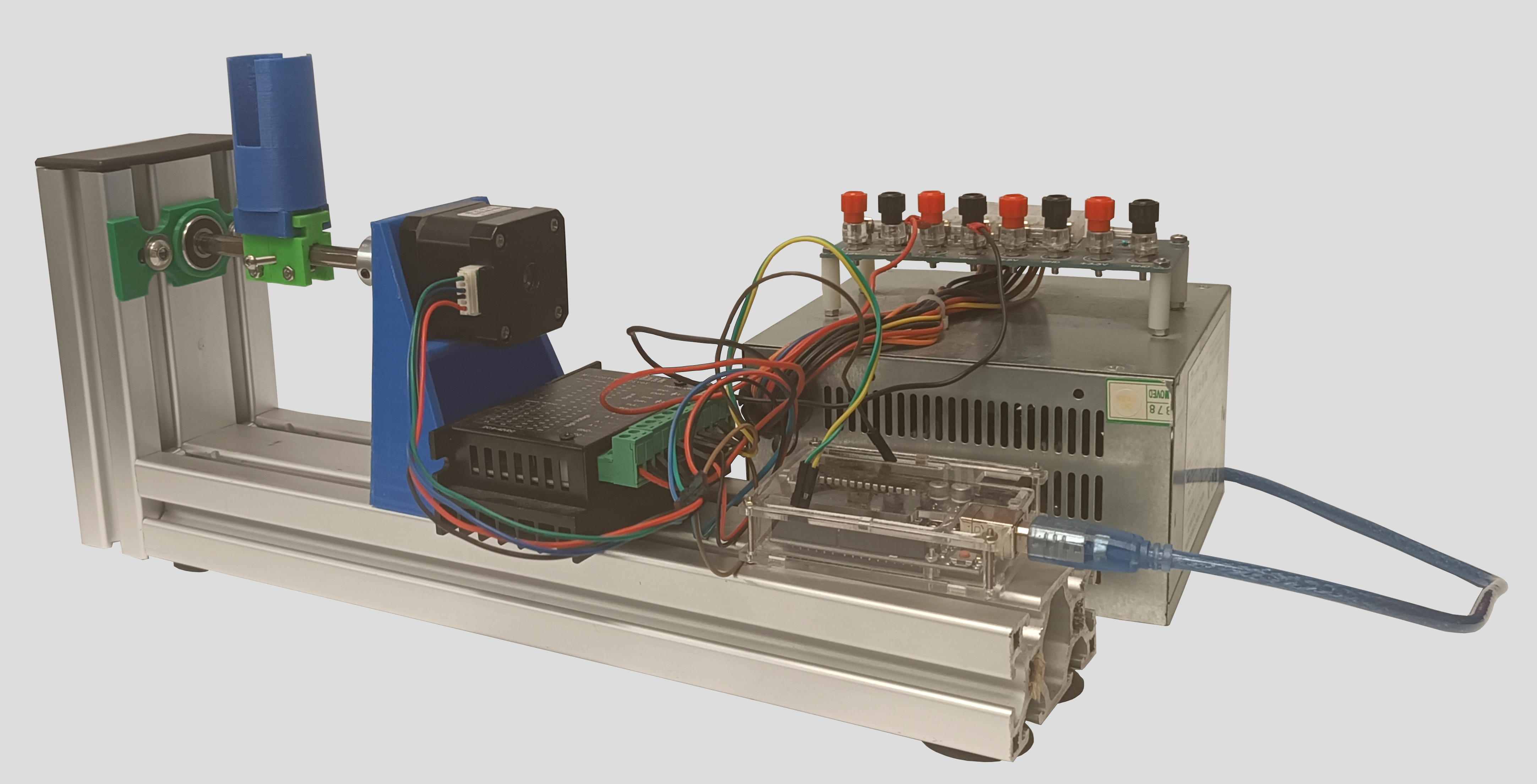


**Fig 8. Testing assembly of CASHo.**

### **Control board**

For the testing assembly the Arduino UNO board was placed inside an acrylic case (Fig 8). For the final mounting: The Arduino UNO board was securely screwed to a PLA wall for a permanent installation (See the video CASHo_homogenization.mp4 at <https://doi.org/10.17605/OSF.IO/ESFWG>). All the STL files for final mounting are in the “Final mounting” folder of the “Components” section in <https://doi.org/10.17605/OSF.IO/ESFWG>.

### TB6600 driver

For the testing setup, the TB6600 driver was placed on the base of the extruded aluminum (Fig 8), without being permanently attached. Switches of the TB6600 were adjusted to 32 micro step per second (6400 pulses per revolution). For the final assembly, the TB6600 driver was securely screwed to a PLA wall, with the heat sink of the driver making direct contact with the base of the extruded aluminum. This implies that the heat sink uses the aluminum base for heat dissipation. (See the video CASHo_homogenization.mp4, at <https://doi.org/10.17605/OSF.IO/ESFWG>).

### Conceptualizing and uploading the firmware

Starting from a position with the tube oriented vertically (original position), the tube is moved to a starting position, corresponding to a 90° rotation clockwise. A cycle is defined as an angular movement of 180° counterclockwise, followed by a return to the starting position (total travel of 360°). A routine consists of an arbitrary number of cycles and includes an additional 90° movement counterclockwise to return to the original position. The firmware logic is illustrated in Fig 9 and the code of firmware is in Homogenizador_v6.ino file, <https://doi.org/10.17605/OSF.IO/ESFWG>).


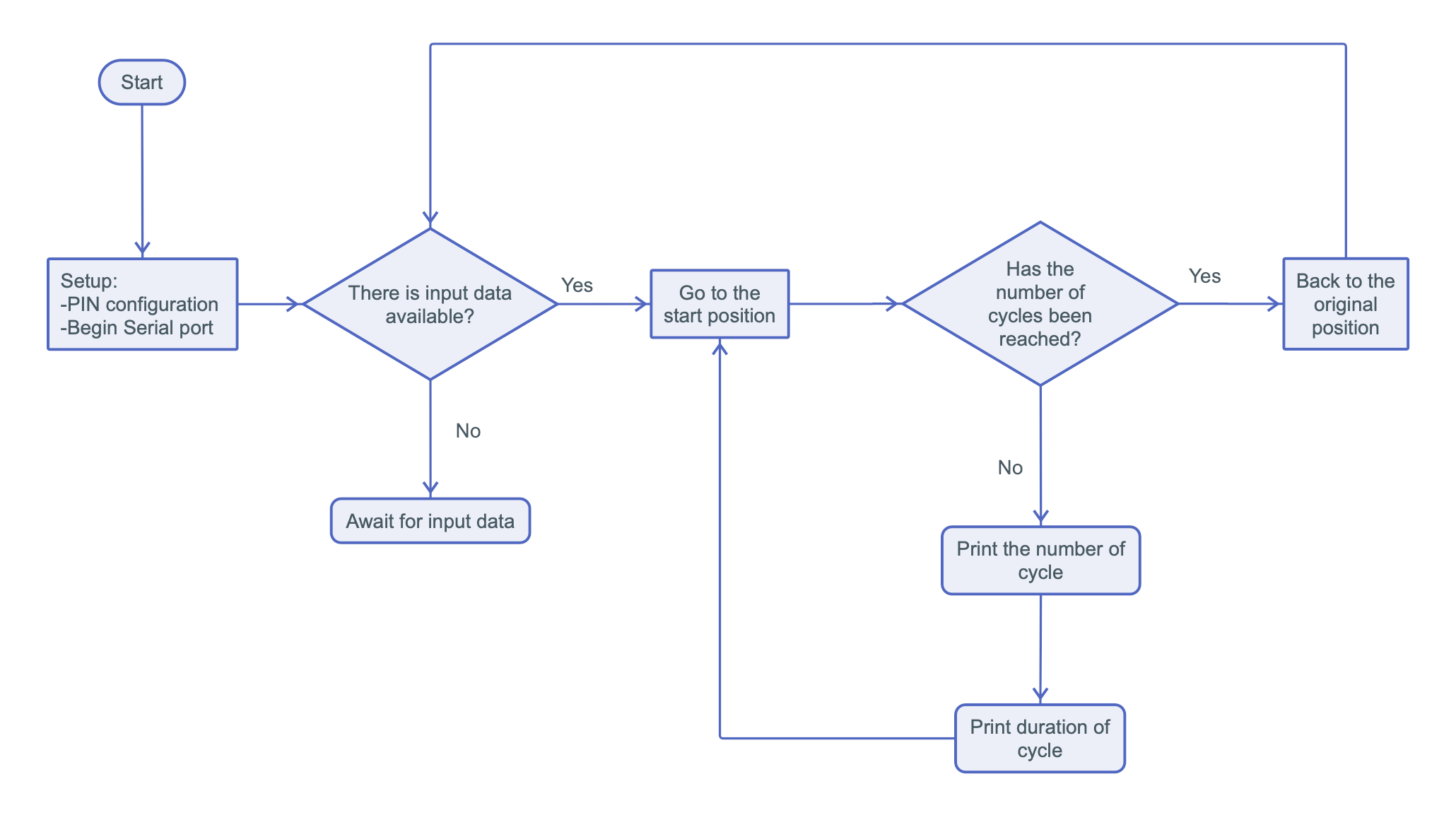


**Fig 9. Firmware logic of CASHo.**

- - **Operation instructions**

Detailed instructions for operation of CASHo are in protocosl.io (<https://dx.doi.org/10.17504/protocols.io.4r3l29ew3v1y/v1>). Here we provide a short version of such instructions:

- - - **Pre-Safety Checks**

Ensure that the aluminum base and all parts are securely fastened. Double-check that all the wiring is properly connected and tightly secured. Verify that the power supply is turned off.

In the computer launch the Arduino IDE, open the file Homogenizer_v6.ino, and verify communication between the IDE and your Arduino UNO board. Upload the firmware is made one time unless the user made changes to the code in Homogenizer_v6.ino.

Ensure that the Tube_holder is in vertical position. This is the original position.

Turn on the power supply to power the stepper motor driver. Hold the Tube_holder with your fingers and ensure that the piece does not move. Place a tube containing diluted semen in the Tube_holder.

Open the serial window in the Arduino IDE and verify that communication is established at 9600 baud. Type "1" or “2” in the input section of the serial window.

The motor shaft will begin to rotate, and the movement of the Tube_holder will be observed.

In the output section of the serial monitor window, you will see the number of cycles set in the routine, as well as the duration of each cycle in seconds (See the Video, CASHo_homogenization.mp4 at <https://doi.org/10.17605/OSF.IO/ESFWG>)
