# Supplementary material for: Development of an open-hardware semen homogenizer and application to serotonin effects on sperm motility
Source: PLoS One. 2025 Dec 5;20(12):e0338399. doi: 10.1371/journal.pone.0338399 (PMC12680138; doi:10.1371/journal.pone.0338399)
Supplement: Attachment — Submitted filename: Response to reviewers.docx [file pone.0338399.s006.docx]

The authors of the manuscript PONE-D-25-30420 “Welcome to the open-hardware sperm quality lab, featuring the new homogenizer: testing serotonin's effects on sperm motility” are grateful with Editor and reviewer's for their valuable suggestions to improve our manuscript. Each one of the comments and suggestions made by Editor and reviewers were addressed, as specified below. Please let me know any other comment or suggestion.

Best regards,

Dr. Andrés Aragón

PONE-D-25-30420

Welcome to the open-hardware sperm quality lab, featuring the new homogenizer: testing serotonin's effects on sperm motility

PLOS ONE

Dear Dr. Aragón,

Thank you for submitting your manuscript to PLOS ONE. After careful consideration, we feel that it has merit but does not fully meet PLOS ONE’s publication criteria as it currently stands. Therefore, we invite you to submit a revised version of the manuscript that addresses the points raised during the review process.

Please submit your revised manuscript by Sep 04 2025 11:59PM. If you will need more time than this to complete your revisions, please reply to this message or contact the journal office at plosone@plos.org. When you're ready to submit your revision, log on to https://www.editorialmanager.com/pone/ and select the 'Submissions Needing Revision' folder to locate your manuscript file.

Please include the following items when submitting your revised manuscript:

A rebuttal letter that responds to each point raised by the academic editor and reviewer(s). You should upload this letter as a separate file labeled 'Response to Reviewers'.

A marked-up copy of your manuscript that highlights changes made to the original version. You should upload this as a separate file labeled 'Revised Manuscript with Track Changes'.

An unmarked version of your revised paper without tracked changes. You should upload this as a separate file labeled 'Manuscript'.

If you would like to make changes to your financial disclosure, please include your updated statement in your cover letter. Guidelines for resubmitting your figure files are available below the reviewer comments at the end of this letter.

If applicable, we recommend that you deposit your laboratory protocols in protocols.io to enhance the reproducibility of your results. Protocols.io assigns your protocol its own identifier (DOI) so that it can be cited independently in the future. For instructions see: https://journals.plos.org/plosone/s/submission-guidelines#loc-laboratory-protocols. Additionally, PLOS ONE offers an option for publishing peer-reviewed Lab Protocol articles, which describe protocols hosted on protocols.io. Read more information on sharing protocols at https://plos.org/protocols?utm_medium=editorial-email&utm_source=authorletters&utm_campaign=protocols.

We look forward to receiving your revised manuscript.

Kind regards,

Sayyed Mohammad Hadi Alavi

Academic Editor

PLOS ONE

Journal requirements:

When submitting your revision, we need you to address these additional requirements.

1. Please ensure that your manuscript meets PLOS ONE's style requirements, including those for file naming. The PLOS ONE style templates can be found at

https://journals.plos.org/plosone/s/file?id=wjVg/PLOSOne_formatting_sample_main_body.pdf and

https://journals.plos.org/plosone/s/file?id=ba62/PLOSOne_formatting_sample_title_authors_affiliations.pdf

**Answer:** Thank you for your recommendations. The manuscript was fully re-structured. Style and file naming now follow the requirements of PloSOne.

2. Thank you for stating the following financial disclosure:

[UNAM, DGAPA, PAPIIT, IN221018, IT201021 and IN224925.].

Please state what role the funders took in the study. If the funders had no role, please state: "The funders had no role in study design, data collection and analysis, decision to publish, or preparation of the manuscript."

If this statement is not correct you must amend it as needed.

Please include this amended Role of Funder statement in your cover letter; we will change the online submission form on your behalf.

**Answer:** Funder had no role. The recommended sentence was included in the Cover Letter.

3. If the reviewer comments include a recommendation to cite specific previously published works, please review and evaluate these publications to determine whether they are relevant and should be cited. There is no requirement to cite these works unless the editor has indicated otherwise.

**Answer:** The recommended previously published works were revised and evaluated.

## Additional Editor Comments:

Dear Prof. Andrés Aragón Martínez

Due date to submit revision: September 20, 2025

Thank you very much for your submission to PLOS ONE. I am glad to inform you that three reviewers with expertise in computer-assisted sperm analysis have reviewed your manuscripts, and provided us with very valuable comments that are appended below. They all highlighted novelty and originality of the present study, however there are many key concerns that require consideration upon revising the MS. Please let me describe in-brief the major comments, while I would appreciate your careful consideration to all of them when you revise the MS.

Some open-software for CASA has been previously introduced and published in the form of a “Research Article”. Under this category, the MS should follow the guidelines according to PLOS ONE. More information is available at:

https://journals.plos.org/plosone/s/what-we-publish

1) In particular, please briefly describe disadvantage and advantage of manual or automated homogenizer in Introduction, and open the idea for developing your device. Introduction in its current version misses a clear aim of your work. Moreover, as reviewer asked for, it is very important to include the rational behind using 5-HT to examine validity of your device. Hence 5-HT stimulates sperm hyper-motility, this could be a good way to examine whether sperm maintain its capacity for hyper-motility during the storage period.

**Answer.** Thank you for your valuable comments. The advantages and disadvantages of manual and automated homogenizers have now been included in the Introduction. The rationale for using 5-HT has also been incorporated. At the respect of hyperactivated motility stimulated by 5-HT we included the following text in Discussion “Capacitation is a key step in the fertilization process (Schmid et al., 2013); thus, long-term semen extenders must prevent capacitation while maintaining motility. Such is the case of the extender used in this study. An event in the capacitation process is the acquisition of hyperactivated motility. Serotonergic communication is related to hyperactivation (Fujinoki, 2011; Sakamoto et al., 2021); however, the *in vitro* development of hyperactivation requires compounds other than 5-HT, such as Ca²⁺, and in the case of boar sperm, additional compounds are needed (Harayama, 2018). Semen extenders may incorporate chelating agents, such as EDTA, which have high affinity for Ca²⁺ (Johnson et al. 2000; Gadea, 2003); thus, hyperactivated motility related to exposure to 5-HT is unlikely. Another factor required to achieve hyperactivation is prolonged incubation (Fujinoki, 2011; Harayama, 2018, Sakamoto et al., 2021), a condition not included in our study. Finally, characteristic motility patterns described for hyperactivated sperm (Suarez, 2003, Fujinoki, 2011; Harayama, 2018) were not observed in our study”.

Gadea J. Review: semen extenders used in the artificial inseminarton of swine. Spanish Journal of Agricultural Research. 2003 June 1;1(2):17–27.

Fujinoki M. Serotonin-enhanced hyperactivation of hamster sperm. Reproduction. 2011;142(2):255–66.

Harayama H. Flagellar hyperactivation of bull and boar spermatozoa. Reprod Med Biol. 2018;17(4):442–8.

Johnson LA, Weitze KF, Fiser P, Maxwell WM. Storage of boar semen. Anim Reprod Sci. 2000;62(1–3):143–72. Gadea J. Review: semen extenders used in the artificial inseminarion of swine. Spanish Journal of Agricultural Research. 2003 June 1;1(2):17–27.

Sakamoto C, Fujinoki M, Kitazawa M, Obayashi S. Serotonergic signals enhanced hamster sperm hyperactivation. J Reprod Dev. 2021;67(4):241–50.

2) Please open “Materials and Methods” next to “Introduction”, at which designs and construction of the hardware, operational instruction, technical issues and limitations, biological test for validation, and statistical analysis are placed.

We are very grateful to you for in-detail description of hardware; however several steps could be moved from the main body into the supplementary data, for instance the required materials and name of suppliers (Table 2).

The method for sperm collection, handling, and spermatozoa motility analysis could be properly described in M&M, as well as statistical analysis for comparison.

**Answer**. Thank you for your recommendations. We have implemented the following changes accordingly:

1) The “Materials and Methods” section has been relocated immediately after the “Introduction”;

2) Table 2, which included the list of required materials and suppliers, has been moved to the supplementary repository, along with the CASHo design files;

3) The procedures for sperm collection, handling, and spermatozoa motility analysis have been relocated and described in detail within the “Materials and Methods” section.

3) Open a result section at which you include the results of sperm density and motility

**Answer**. Thank you for your recommendation. A section with results of sperm concentration and motility was added to the Results.

4) The MS needs “Discussion” in which the advantage of the hardware should be compared with existed manual or other type of homogenizers. In this section, the results of biological test could be described. In this latter case, please describe whether 5-HT stimulation of spermatozoa hyper-motility was maintained or any other physiological system that 5-HT acts on spermatozoa motility. Showing statistical difference is essential for Figure 10.

**Answer**. Thank you for your comments. A Discussion section was added; in this section advantage of CASHo over devices available in the market and open-source are discussed. In the Discussion section the results of stimulation of sperm with 5-HT are also discussed. Factors needed for activated motility are mentioned. In Figure 10 (now Figure 1) the significant changes are shown.

5) Please avoid redundant of results. I found data in Table 3 are identical to those shown in Figure 11.

**Answer**. Thank you for your observation. Table 3 was deleted, and a new figure was added to show the proportions of sperm in each subpopulation and treatment (as reviewer 2 requested). Significant differences are included in the text and in heatmaps.

6) Please reduce number of figures with making together some current figures. Another example is to move individual variation in sperm density (Figure 10) to supplementary data, and keep panel showing the mean values (bottom panel).

**Answer**. Thank you for your valuable comment. We move Figure 1-9 to a document called CASHo_supplementary_details.pdf, which was moved to the repository <https://osf.io/esfwg/>, at the Supplementary information section.

7) Please uniform “biological terms” through the MS, and edit the English before re-submission.

**Answer**. Thank you for your recommendation. The “biological terms” have been carefully revised and standardized throughout the manuscript. In addition, the English language has been edited by a professional service with native English speakers.

8) Please find examples of some open CASA software published previously, one is from PLoS Comput Biol:

Alquézar-Baeta C, Gimeno-Martos S, Miguel-Jiménez S, Santolaria P, Yániz J, Palacín I, et al. (2019) OpenCASA: A new open-source and scalable tool for sperm quality analysis. PLoS Comput Biol 15(1): e1006691. https://doi.org/10.1371/journal.pcbi.1006691

Butts, I.A., Ward, M.A., Litvak, M.K., Pitcher, T.E., Alavi, S.M., Trippel, E.A. and Rideout, R.M. (2011) Automated Sperm Head Morphology Analyzer for Open-Source Software. Theriogenology, 76, 1756-1761.

http://dx.doi.org/10.1016/j.theriogenology.2011.06.019

Wilson-Leedy JG, Ingermann RL, (2006) Development of a novel CASA system based on open source software for characterization of zebrafish sperm motility parameters, Theriogenology 67(3):661-72. https://doi.org/10.1016/j.theriogenology.2006.10.003

**Answer**. Thank you for providing examples of open-source CASA software. Our manuscript does not describe the development of a new CASA software. Instead, it reports the use of an existing open-source plugin (Rivas et al., 2022), which is based on previously published tools. These foundational publications were properly cited in Rivas et al., (2022): “We looked at three to four fields per sample and analysed the image sequences using ImageJ software version 1.50d (Rasband, 1997) and the plugin developed by Wilson-Leedy and Ingermann (2007), later modified by Giaretta et al. (2017). We further adapted the plugin (CASA_bgm) for boar sperm by adjusting the parameter settings.”

Giaretta, E., Munerato, M., Yeste, M., Galeati, G., Spinaci, M., Tamanini, C., Mari, G., & Bucci, D. (2017). Implementing an open-access CASA software for the assessment of stallion sperm motility: Relationship with other sperm quality parameters. *Animal Reproduction Science*, *176*, 11–19. <https://doi.org/10.1016/j.anireprosci.2016.11.003>

Rivas, C. U., Ayala, M. E., & Aragón, A. (2022). Effect of various pH levels on the sperm kinematic parameters of boars. South African Journal of Animal Science, 52(5), 693–704. <https://doi.org/http://dx.doi.org/10.4314/sajas.v52i5.13>

Wilson-Leedy JG, Ingermann RL, (2006) Development of a novel CASA system based on open source software for characterization of zebrafish sperm motility parameters, Theriogenology 67(3):661-72. <https://doi.org/10.1016/j.theriogenology.2006.10.003>

Once again, I appreciate choosing PLOS ONE for your work, and I hope our review provide you with valuable comments on your research.

With very best regards

Hadi Alavi

## Reviewer #1:

The manuscript “Welcome to the open-hardware sperm quality lab, featuring the new homogenizer: testing serotonin's effects on sperm motility” is aims to design, construct, and validate an objective homogenizer for boar semen doses.
I appreciate the importance of studying the effects of semen homogenization in the laboratory, and at first glance, your manuscript may provide interesting data.
However, we have some concerns with the general contents and the presentation.

***Major points***
How many ejaculates from each boar were used in Experiment 1?

**Answer**. Tank you for your inquiry. For the experiment evaluating sperm concentration at different number of cycles, one semen sample from each of five boars was used. Five aliquots were taken from each semen sample. The five aliquots from each sample were individually processed using 5, 10, 15, 20, or 25 homogenization cycles with CASHo, one aliquot per cycle level. This information was added to the manuscript.

Semen collection records with lower sperm motility were excluded? What was the threshold criterion for total motility? The collection periods before and during the experiment did not account for the spermatic wave?

**Answer**. Thank you for your inquiries. The five boars used in this study were semen donors from the Liquid Gene Transfer Center (LGTC) at the CEIEPP, Faculty of Veterinary Medicine and Animal Science, UNAM. These animals were selected based on proven fertility and are routinely used in genetic dissemination programs for regional pig producers, as well as for pig production at the center’s experimental farm.

All donor boars follow a strict management protocol, which includes weekly semen collections. This schedule ensures consistent semen quality in terms of volume and concentration. Collections with progressive motility below 80% or with more than 15% morphological abnormalities are routinely discarded. Therefore, only ejaculates meeting these quality criteria were used in the present experiment. Samples were subsequently transported and evaluated before and after dilution using a commercial extender (1:1 volume-to-volume), as described in the manuscript. This information was added to the Material and Methods.

Regarding the reviewer’s mention of the “spermatic wave,” we assume this refers to the spermatogenic wave—that is, the orderly and cyclic progression of spermatogenesis along the seminiferous tubules. The collection schedule used in this study was designed to ensure uniform sperm quality across time and was not specifically synchronized with the seminiferous epithelial cycle, as supported by previous work (Rodríguez-Martínez et al, 2024; Parrish et al., 2017).

Rodriguez-Martinez H, Martinez-Serrano CA, Alvarez-Rodriguez M, Martinez EA, Roca J. Reproductive physiology of the boar: What defines the potential fertility of an ejaculate? Anim Reprod Sci. 2024;269:107476.

Parrish JJ, Willenburg KL, Gibbs KM, Yagoda KB, Krautkramer MM, Loether TM, et al. Scrotal insulation and sperm production in the boar. Mol Reprod Dev. 2017;84(9):969–78.

The authors analyze sperm kinematic variables and kinematic patterns in boar semen subpopulations. However, data on total and progressive motility (%) are not presented in the study. Since total and progressive motility are fundamental variables for semen analysis and the production of semen doses- whether fresh, chilled, or even frozen- the authors should include these results in the manuscript. This would help determine the effect of homogenization on these variables and address the current lack of evidence regarding basic motility parameters, as opposed to only advanced (kinematic) variables.
Can the authors provide data on the morphology of boar sperm? The authors could provide results on the effect of homogenization on sperm morphology, particularly regarding head and midpiece defects such as decapitated spermatozoa, in order to assess the impact of homogenization on the incidence of sperm morpho-anomalies. I agree with the authors that sperm quality is important to establish its relationship with male fertility; therefore, this information is essential for the study.

**Answer**. Thank you for your valuable comments. Data on total and progressive motility have now been included in the manuscript and are discussed accordingly.

Unfortunately, we do not have data on sperm morphology. However, we did not observe decapitated spermatozoa in the analyzed samples. This result is consistent with the nature of the homogenization method employed, which involves relatively slow forward and backward movements. This gentle mixing approach likely minimizes mechanical damage to the spermatozoa.

A demonstration of the hand-operated homogenization technique can be found in the video Hand_operated_homogenizer.mp4 available in the video section of the following repository: <https://doi.org/10.17605/OSF.IO/ESFWG>, which our method is based on.

The experiment on serotonin is not clearly aligned with the central objective of the study (The goal of this study was to design, construct, and validate an objective homogenizer for boar semen doses). The authors should justify the inclusion of this experiment in the manuscript and explain how it relates—if at all—to the main objective of the article.

**Answer**. Thank you for your valuable comment. Justification of inclusion of experiment, where effects of 5-HT and cycles were investigated, is now included.

Finally, I believe this study provides valuable information regarding semen analysis methodology in the laboratory.

**Answer**. We are very grateful with your kind comment.

The largest issue with this study is that the experimental design is unclear. If it is the latter, then the differences observed may simply be differences between boars, and not between cycles. There is no model, and the authors do not provide one in their study.

**Answer**. Thank you for your valuable comment. The experimental design is now clearly described in the revised version of our manuscript. The effect of the boar was explicitly included as a random factor in the statistical model, as detailed in the Statistics section.

Can the authors indicate a clear and summarized conclusion of this work?

**Answer**. Thank you for your inquiry. A section of Conclusions was included.

## Reviewer #2:

Review Report
Title: Welcome to the open-hardware sperm quality lab, featuring the new homogenizer: testing serotonin's effects on sperm motility

***Overall assessment***
This manuscript presents a novel, open-source device (CASHo) for objective semen homogenization, and investigates its application in studies on serotonin’s effects on boar sperm motility. The technical innovation is commendable and valuable for labs with limited resources. However, the current structure of the manuscript overly emphasizes hardware details at the expense of biological analysis and interpretation.
The experimental design is of interest, and the identification of sperm kinematic subpopulations is a strong biological element. Yet the manuscript needs significant reorganization and revision to meet the scientific standards expected by PLOS ONE. Below are major and minor suggestions to improve clarity, structure, and scientific impact.

***Major comments***
Restructure the manuscript – move detailed hardware descriptions to supplementary materials
The current version devotes excessive space to construction details (e.g., STL file descriptions, wiring diagrams, firmware logic). This level of detail is valuable, but it should be moved to Supplementary Materials. The main manuscript should include only a concise overview highlighting why CASHo is different, how it improves over existing methods, and what the key design parameters are.

**Answer**. Thank you for your valuable comments. The STL file descriptions, wiring diagrams, and firmware logic have been moved to a supplementary file named CASHo_Supplementary_Details.pdf. This file has been uploaded to our Open Science Framework (OSF) project page at <https://osf.io/esfwg/>, specifically in the supplementary information section, accessible directly via <https://osf.io/aydk4/>.

Retain calibration data as an experimental foundation

The experiment determining the number of cycles needed for full sperm homogenization is valuable and scientifically necessary. This should remain in the main manuscript, clearly defined as a preliminary calibration step for establishing the operational definition of “objective homogenization” prior to biological experiments. A brief rationale should be included to explain why 5 cycles was selected as the standard condition.

**Answer**. Thank you for your valuable comments. The experiment determining the number of cycles required for full semen homogenization has been retained in the revised manuscript and is now explicitly presented as a preliminary calibration step. This calibration establishes the operational definition of “objective homogenization” prior to conducting the biological experiments. Based on the results of this experiment, sperm concentration stabilized after five cycles, with no further significant changes observed at higher cycle numbers; therefore, five cycles was selected as the standard condition for all subsequent assays.

Elevate the biological relevance – center the subpopulation analysis
The most biologically meaningful part of the study is the identification of kinematic sperm subpopulations and their distinct responses to serotonin. This section should be expanded and moved into the main Methods and Results sections. Clearly explain the PCA and clustering strategy and provide justification for the choice of three clusters.

**Answer**. Thank you for your comments. The analysis of kinematic subpopulations is now described in detail in the Materials and Methods and Results sections. The PCA approach and the rationale for selecting three clusters have also been included and explained in the revised manuscript.

Analyze all subpopulations and whole sample
Only one subpopulation (Subpop 2) is presented in the main text. The rationale behind is not clear, nor, I think “biologically relevant”. Please include the full analysis for all three subpopulations and also show results for the entire unsegmented sperm population. This would allow readers to compare how much information is lost or gained by using subpopulation analysis versus pooled data.

**Answer**. Thank you for your valuable comments. A complete analysis of all three kinematic subpopulations has been added to the revised manuscript. In addition, the biological insights gained through subpopulation analysis compared to the use of pooled (average) data are discussed.

Report the proportional distribution of subpopulations
It would strengthen the manuscript to include percentage representation of each cluster across all experimental groups (you have provided the numbers of sperm in each subpopulation, just add the percentage along with it). This could help detect whether serotonin or homogenization changes the relative size of clusters (e.g., shift from more motile to less motile populations), which is biologically significant.

**Answer**. Thank you for your comment. A mosaic plot illustrating the proportional distribution of sperm across clusters, treatments, and number of homogenization cycles has been included in the revised manuscript. This visualization allows for the detection of shifts in subpopulation structure under different experimental conditions, as suggested.

Consider integrating additional sperm parameters.
If feasible, consider combining subpopulation data with other available sperm quality markers such as membrane integrity, mitochondrial activity, or acrosome status. This would deepen the biological understanding of each subpopulation and potentially reveal phenotype-function correlations. This could be addressed in additional experiments or simply highlighted as an important future direction—demonstrating the broader utility of both the CASHo device and subpopulation analysis, well beyond serotonin-related studies.

**Answer**. Thank you for your valuable comments. We have now emphasized the relevance of additional sperm quality markers—such as membrane integrity, mitochondrial activity, and acrosome status—in relation to kinematic subpopulations and the potential applications of the CASHo device. This connection has been highlighted as an important future direction in the revised manuscript.

At line 444 you stated that subpopulation 2 is “biologically relevant”. I think this is not correct. Relevant to what? All sperm clusters were biological samples. Dividing them into three subclasses allows for a better understanding of the effect of serotonin—highlighting which subgroup is most sensitive to the treatment and which is most resistant. That’s biology: individual variation grouped into clusters or subpopulations. This approach is already interesting in itself, but it would be even more informative if the percentage of each subpopulation (from the most affected to the most resistant) was presented. Ideally, these data could be combined with other biological parameters of the sperm, such as membrane stability tests, mitochondrial status, etc. This would shed more light on the data and might allow for a higher-level interpretation of subpopulation characteristics—for example, by showing correlations between the proportion of spermatozoa with unstable membranes and the subpopulation most affected by serotonin (or perhaps another trend—I’m just speculating).

**Answer:** Thank you for your valuable comments. The importance of velocity in fertilization has been previously reported (Donnelly et al., 1998); therefore, we initially focused on cluster 2, which included spermatozoa with the highest values of VCL (point-to-point velocity). However, all clusters are now included in the Results and discussed accordingly. We also emphasize the importance of considering additional parameters in future studies, which we highlight as directions for further research.

Donnelly ET, Lewis SE, McNally JA, Thompson W. In vitro fertilization and pregnancy rates: the influence of sperm motility and morphology on IVF outcome. Fertil Steril. 1998 Aug;70(2):305–14.

Clarify language and improve precision
The manuscript often uses vague or non-scientific terms. For example, Line 394 uses the word "observed" in a way that lacks analytical meaning. I recommend replacing it with a phrase such as “proven to be effective” or “shown to significantly alter 5 cycles”.

**Answer**. Thank you for you valuable comment and suggestion. The word “Observed” was replaced with “proven to be effective” accordingly.

**Minor comments**
Abbreviations: Ensure all abbreviations (e.g., VCL, STR, etc.) are defined at first use.

**Answer**. Thank you for your comment. All abbreviations have now been defined at their first occurrence in the manuscript.

**Conclusion**
This is a promising manuscript that combines bioengineering with reproductive biology, but a major revision is needed to shift focus from the technical construction to biological insight. The revised version should be more biologically oriented, clearly structured, and analytically precise.

**Answer:** Thank you for your valuable comments. The manuscript has been fully restructured. In this revised version, we aimed to maintain the link between technical construction, bioengineering, and reproductive biology; however, greater emphasis has been placed on the biological aspects of the study.

## **Reviewer #3:**

The reviewed manuscript describes the development and validation of an open-hardware sperm homogenizer for boar semen, including a demonstration of its impact on sperm motility under several mixing regimes and after serotonin treatment. The topic is timely and relevant for laboratories working in reproductive biology and animal reproduction, especially in resource-limited settings. The authors present a high level of technical detail and reproducibility, including design files and firmware.
Nevertheless, the manuscript requires significant clarification, reframing, and improvement in presentation before it can be considered for publication.
Below are some major and minor comments for revision.

***Major comments***
**Title of the manuscript:**
The title “Welcome to the open-hardware sperm quality lab…” is catchy but somewhat misleading, as the manuscript primarily describes the development and validation of a single custom device, rather than a comprehensive laboratory platform. I recommend revising the title to focus on the sperm homogenizer (or mixer) development.
The title also places serotonin effects on equal level with the technical innovation. This is especially evident in the short title. However, the serotonin experiment appears to serve mainly as a demonstration of the device’s utility, rather than as a hypothesis-driven biological investigation. The serotonin aspect could likely be omitted from the title without loss of clarity.

**Answer**: Thank you for your valuable comments. The manuscript has been revised to emphasize both the effects of serotonin and the open-hardware device developed in this study. Our intention is not to present a broad laboratory platform, but rather to describe and validate a single device with direct biological application. We also believe that the current title includes the essential keywords needed for readers to identify and locate our work. Therefore, we respectfully request to retain the original title.

**Scientific justification for the serotonin experiment:**
The rationale for combining serotonin treatment with over-homogenization is insufficiently substantiated. Why would over-homogenization interact with serotonin exposure in a way that demonstrates the value of the device? It is unclear whether this experiment was intended primarily as proof-of-concept or if it follows a mechanistic hypothesis. Please clarify the purpose and reframe the Results section if the goal is demonstration only.

**Answer**. Thank you for your valuable comments and inquiry. We have now included a clearer rationale explaining the experimental design in which spermatozoa were exposed to serotonin under different numbers of homogenization cycles. The aim was to evaluate both the biological response to serotonin and the potential interaction with mechanical stress, thereby demonstrating the utility of the device. The All sections has been revised accordingly to make this purpose explicit.

Additionally, the term over-homogenization appears without justification. What exactly is meant by this term? How does it occur? What are its biological consequences? How can it be measured or prevented? Is it a species-specific phenomenon? These aspects should be discussed more explicitly.

**Answer**. Thank you for your inquiries. After establishing an objective definition of semen homogenization, we investigated the effects of increasing the number of cycles on sperm motility, once this baseline level of homogenization had been achieved. Biological consequences of over-homogenization are presented in Results and Discussed accordingly

**Comparison with existing commercial devices:**
The authors state that commercially available orbital or swing shakers cannot replicate manual hand motion and that this is important for semen handling. However, this claim lacks direct evidence or comparative testing. It would strengthen the study to cite published data or provide pilot comparisons showing, for example, that the motion replicated by the device results in better sperm distribution (e.g., lower variance in concentration or reduced damage to spermatozoa) compared to commercial mixers.

**Answer**. Thank you for this important comment. The current version of the manuscript does not include comparative data between our device and commercially available equipment, as we did not have access to such equipment during the study period. However, published studies have shown that mechanical homogenization, using commercial devices, increased the production of reactive oxygen species (ROS), which in turn may compromise sperm membrane integrity, motility, and viability (Menegat eta l., 2017; Schulze et al., 2018; Tamanini et al., 2022; Paschoal et al., 2021). When hand was compared with mechanic homogenization alterations in sperm motility were observed (Broekhuijse et al., 2011). Based on this rationale, it is reasonable to hypothesize that manual or gentle homogenization methods may have fewer detrimental effects on sperm quality. The previous information is now included in the mannuscript.

Broekhuijse MLWJ, Šoštarić E, Feitsma H, Gadella BM. Additional value of computer assisted semen analysis (CASA) compared to conventional motility assessments in pig artificial insemination. Theriogenology. 2011;76(8):1473-1486.e1.

Menegat MB, Mellagi APG, Bortolin RC, Menezes TA, Vargas AR, Bernardi ML, et al. Sperm quality and oxidative status as affected by homogenization of liquid-stored boar semen diluted in short- and long-term extenders. Anim Reprod Sci. 2017;179: 67–79. doi:10.1016/j.anireprosci.2017.02.003

Schulze M, Bortfeldt R, Schäfer J, Jung M, Fuchs-Kittowski F. Effect of vibration emissions during shipping of artificial insemination doses on boar semen quality. Animal Reproduction Science. 2018;192: 328–334. doi:10.1016/j.anireprosci.2018.03.035

Tamanini MSC, dos Santos G, Leal LA, Wolf LM, Schulze M, Christ TS, et al. Impact of agitation time of boar semen doses on sperm traits in short- and long-term extenders. Animal Reproduction Science. 2022;247: 107159. doi:10.1016/j.anireprosci.2022.107159

Paschoal AFL, Luther A-M, Jakop U, Schulze M, Bortolozzo FP, Waberski D. Factors influencing the response of spermatozoa to agitation stress: Implications for transport of extended boar semen. Theriogenology. 2021;175: 54–60. doi:10.1016/j.theriogenology.2021.08.033

**Terminology and clarity of processes:**
While the manuscript consistently uses the term homogenization, it does not clearly distinguish the separate processes involved in semen handling. For clarity, I suggest distinguishing: 1. Re-suspension of spermatozoa (to redistribute settled cells); 2. Mixing of soluble additives such as serotonin; 3. Incubation time to allow interactions to occur.

**Answer**. Thank you for your valuable comments. The processes of re-suspension, mixing of soluble additives, and incubation time are now clearly distinguished in the manuscript.

Additionally, the term homogenization can imply mechanical disruption of cells, which may not be what the authors intend. Please clarify what exactly is meant in your usage of this term, and why homogenization is preferred over alternatives such as mixing or re-suspension.
The manuscript also uses word “sperm” inconsistently, sometimes referring to cells and sometimes to the fluid. Consider using spermatozoa when referring to the cells and semen when referring to the suspension.

**Answer**. Thank you for your valuable comments. The use of the term homogenization has now been clarified and justified in the manuscript. In addition, the terminology has been carefully revised to ensure consistent use of spermatozoa when referring to the cells and semen when referring to the suspension.

**Data presentation and statistical analysis:**
While the manuscript provides a relatively detailed description of the statistical workflow, particularly for the motility subpopulation analysis, some key aspects remain unclear. Please clarify how biological replicates (i.e., semen samples from individual boars) were handled in the statistical models. If the same semen sample was split and subjected to multiple treatment conditions (e.g., different mixing cycles), this constitutes a repeated-measures design, and a repeated-measures ANOVA (or mixed model) may be more appropriate. If a standard ANOVA was used, please justify the assumption of independence and clarify whether boar ID was included as a blocking or random factor. Additionally, reporting effect sizes or confidence intervals alongside p-values would improve transparency and interpretability.
**Answer**. Thank you for your comments. For the experiment evaluating sperm concentration at different numbers of homogenization cycles, five aliquots were taken from each semen sample. Each aliquot was individually processed with 5, 10, 15, 20, or 25 cycles using the CASHo device, with one aliquot assigned to each cycle level.

For the serotonin exposure experiment, ten aliquots were obtained from each semen sample: five served as controls (Vh group) and five were treated with serotonin (5-HT group). As in the previous experiment, each aliquot was processed with a specific number of cycles (5, 10, 15, 20, or 25), such that one aliquot per treatment group was assigned to each cycle level. The effect of 5-HT was compared with the Vh group at five cycles (the objective homogenization condition) using a Student’s t-test. For the over-homogenization conditions (10–25 cycles), a mixed-effects model was applied. Briefly, the model for each motility parameter within each cluster included the fixed effects of treatment (Vh or 5-HT), number of homogenization cycles (10, 15, 20, or 25), and their interaction. Boar identity was incorporated as a random effect to account for inter-individual variability.

This approach allowed us to evaluate the combined influence of treatment and mechanical processing on sperm motility while controlling for biological variation among boars. The description of the statistical model has now been added to the Statistics section.

**Language and style:**
The manuscript contains numerous grammatical issues, inconsistent phrasing, and unclear terminology. For example, the phrase “homogenization of serotonin” is scientifically inaccurate. A thorough English language and grammar review is strongly recommended to improve clarity and readability, examples are given in minor comments.
**Answer**. Thank you for your valuable comments. The language has been thoroughly revised, and the final version of the manuscript was professionally edited by a commercial service with native English-speaking editors.

***Minor comments:***
Line 28: “Correct number” — consider using “predictable number” or “consistent number” instead.

**Answer**. Thank you for your suggestions. “Correct number” was replaced with “consistent number”.

Line 39: “The effects of homogenization and overhomogenization…” — consider rephrasing as “potential over-homogenization.”

**Answer**. Thank you for your comment. The text was modified accordingly.

Line 45: The meaning of “Objective homogenization” is unclear

**Answer**. Thank you for your comment. By “objective homogenization,” we refer to a procedure involving a defined number of mixing cycles after which sperm concentration no longer changes. This operational definition is provided in the penultimate paragraph of the Introduction.

Line 53: Revise for clarity — suggest: “sperm motility is evaluated using a customized open-source computer-assisted sperm analysis system (CASA) [1].”

**Answer**. Thank you for your suggestion. The text was modified accordingly.

Line 54: “The evaluation of sperm concentration and vertical velocity is evaluated…” — rephrase to avoid redundancy.

**Answer**. Thank you for you observation. The text was replaced with “and sperm concentration and vertical velocity are evaluated in the UPSPERM”.

Line 65: “Gentle homogenization is necessary because vigorous agitation can damage cells” — this is a key claim and should be supported with a reference.

**Answer**. Thank you for your recommendation. We have added supporting information and references to substantiate this claim. The following sentence was included in the revised manuscript:

“For boar sperm, viability and acrosome integrity have been shown to correlate negatively with vibration emissions during transport (Schulze et al., 2018), while mitochondrial activity was reduced after agitation in an orbital shaker at 70 RPM (Tamanini et al., 2022). Additionally, vibration-induced stress increased ROS levels (Paschoal et al., 2021) and reduced tyrosine phosphorylation (Wang et al., 2024).”

Schulze M, Bortfeldt R, Schäfer J, Jung M, Fuchs-Kittowski F. Effect of vibration emissions during shipping of artificial insemination doses on boar semen quality. Animal Reproduction Science. 2018;192: 328–334. doi:10.1016/j.anireprosci.2018.03.035

Tamanini MSC, dos Santos G, Leal LA, Wolf LM, Schulze M, Christ TS, et al. Impact of agitation time of boar semen doses on sperm traits in short- and long-term extenders. Animal Reproduction Science. 2022;247: 107159. doi:10.1016/j.anireprosci.2022.107159

Paschoal AFL, Luther A-M, Jakop U, Schulze M, Bortolozzo FP, Waberski D. Factors influencing the response of spermatozoa to agitation stress: Implications for transport of extended boar semen. Theriogenology. 2021;175: 54–60. doi:10.1016/j.theriogenology.2021.08.033

Wang S, Zeng X, Liu S, Hoque SAM, Min L, Ding N, et al. Vibration Emissions Reduce Boar Sperm Quality via Disrupting Its Metabolism. Biology. 2024;13: 370. doi:10.3390/biology1306037

Line 74: “Proprietary hardware is multi-purpose…” and “…specifically intended.”

**Answer**. Thank you for your recommendations. The text was modified accordingly.

Line 78: VAT is already a tax; “plus tax and VAT” is redundant.

**Answer**. Thank you for your observation. The text was modified accordingly.

Line 78: revise to “a programmable rotary agitator.”

**Answer**. Thank you for your suggestion. The text was modified accordingly.

Line 84: Clarify why “gentle” mixing is technically non-achievable with the referenced tool.

**Answer**. Thank you for your comment. The flask-shaker wrist-action device offers both “vigorous” and “gentle” mixing depending on the selected speed (ranging from 80 to 800 RPM), but it operates at a fixed inclination of approximately 5 degrees. This limited inclination angle, combined with the rotational mechanism, may increase vibration and mechanical stress on the cells—even at lower speeds (e.g., 80 RPM are 1.3 RPS, which is still higher than typical hand mixing). These mechanical effects can potentially compromise sperm motility and viability. Moreover, according to the manufacturer, the device is intended for prolonged and continuous shaking, which is not ideal for handling sensitive biological samples such as semen. The sentence was re-structured to include this information.

Line 86: Transition needed. For example: “One of the key applications of sperm homogenization is during sperm sample storage.” Or similar.

**Answer**. Thank you for your suggestion. We added the transition accordingly.

Line 87: “Commonly at 17°C” — is this is species-specific, I guess; please clarify.

**Answer**. Thank you for you request. The recommended temperature for storing boar semen used on-farm artificial insemination programs is 16–17°C. This allows us to maintain viable semen with acceptable fertility for up to 7 days of storage, using commercial long-term extenders. In our study, we maintained 17°C as it appears to have slight benefits on the metabolic activity and membrane integrity of boar spermatozoa (Deziekonska et al. 2013). We included the previous information in the Material and methods, subsection Semen samples.

Dziekońska A, Fraser L, Majewska A, Lecewicz M, Zasiadczyk Ł, Kordan W. Effect of commercial long-term extenders on metabolic activity and membrane integrity of boar spermatozoa stored at 17 degrees C. Pol J Vet Sci. 2013;16(3):517–25.

Line 87: “Storage of samples leads to sedimentation of sperm cells, which must be homogenized before use.” Suggest rephrasing for clarity.

**Answer**. Thank you for your suggestion. The text was replaced with “During storage, sperm cells tend to sediment, so the samples must be homogenized before use.”

Lines 91–107: I suggest including references to positive consequences of resuspension of sperm during hypothermal storage reported e.g. in fish sperm.

**Answer**. Thank you for your suggestion. We have now included a reference supporting the benefits of sperm resuspension during hypothermal storage. For example, Love et al. (2012) demonstrated that daily resuspension of cooled equine semen helps preserve sperm longevity and quality.

Love CC, Blanchard TL, Varner DD, Brinsko SP, Voge J, Bliss S, et al. Effect of daily semen centrifugation and resuspension on the longevity of equine sperm quality following cooled storage. Theriogenology. 2012;77: 1911–1917. doi:10.1016/j.theriogenology.2012.01.010

Line 110: Why is the mentioned rotator not suitable for semen? Please elaborate.

**Answer**. Thank you for your question and comment. The device has two hardware versions:

The first operates with continuous 360° rotation and lacks velocity control. This tends to cause cell accumulation at a specific point in the tube and increases the formation of air bubbles, both of which can negatively affect sperm quality. The second version uses a programmable microcontroller to control the rotation angle, but operates with a limited inclination range. This configuration can increase vibration and mechanical stress on the cells, potentially compromising motility and viability.

Additionally, this type of equipment is primarily designed for mixing chemical solutions, not for handling sensitive biological materials such as semen. Finally, both hardware versions rely on DC motors, which require additional electronic components to regulate velocity and angular movement with precision. The previous information was added to the manuscript.

Lines 122–124: The sentence should be rephrased for clarity.

**Answer**. Thank you for you suggestion. The sentence was rephrased to “Given the absence of a standard operational definition, we define full homogenization of the sperm suspension as the point at which further mixing no longer produces changes in the measured sperm concentration.”

Lines 136–137: Why should hand motion be replicated? Are there comparative studies on mixing efficiency or biological outcomes?

**Answer**. Thank you for your questions. Hand motion was replicated because it is a widely used practice in semen handling, allowing for gentle and controlled homogenization of the sample. This method helps minimize the formation of air bubbles, cell stress, and turbulence, all of which can negatively affect sperm quality.

There is a report where semen homogenization by hand was compared with mixing using a vortex and alterations on sperm motility were found (Broekhuijse et al., 2011); this information is now included in Discussion. Hand mixing remains a standard empirical technique in many laboratories. In our work, we aimed to design a system that closely mimics this commonly used manual procedure. A demonstration of the reference hand movement is available in the video Hand_operated_homogenizer.mp4, found in the video section of our OSF repository: <https://doi.org/10.17605/OSF.IO/ESFWG>.

Broekhuijse MLWJ, Šoštarić E, Feitsma H, Gadella BM. Additional value of computer assisted semen analysis (CASA) compared to conventional motility assessments in pig artificial insemination. Theriogenology. 2011;76(8):1473-1486.e1.

Lines 146–147: The device is said to work for “other cell types” — it would be more accurate to say “other cell suspensions,” and clarify that technical parameters can be adjusted, though effectiveness should be obviously tested case by case.

**Answer**. Thank you for the suggestion. We agree that "other cell suspensions" is more accurate. The sentence has been revised accordingly to reflect this, and we have added a clarification regarding the need to adjust technical parameters and to validate effectiveness on a case-by-case basis. The new sentence say: “Furthermore, it can also be adapted for homogenizing other cell suspensions, although technical parameters may require adjustment, and effectiveness should be validated on a case-by-case basis.”

Line 354: Use “animal ID” instead of “boar.”

**Answer**. Thank you for the comment. “boar” was replaced with “boar ID”.

Line 358: Rephrase for clarity e.g., “Sperm concentration was higher in samples stored for less than 5 hours post-collection than after 24 hours.”

**Answer**. Thank you for your suggestion. The text “The lower sperm concentration was found at 0 cycles for less than 5 hours and after 24 hours (Fig 10); but sperm concentrations was greater at less than 5 hours than after 24 hours (1.8e8 and 0.3e8 spz/ml, respectively)” was replaced with “The lower sperm concentration was found at 0 cycles for less than 5 hours and after 24 hours (Fig 10); nonetheless, samples stored for less than 5 hours showed a higher concentration (1.8 × 10⁸ spz/ml) than those stored for 24 hours (0.3 × 10⁸ spz/ml).”

Line 382: Should be “We are interested in investigating…”

**Answer**. Thank you for the observation. The text was replaced accordingly.

Line 382: Please clarify the rationale for testing serotonin in the context of device development.

**Answer**. Thank you for you r comment. Rationale for testing serotonin and cycles is now included.

Line 384: Define and justify the concept of “over-homogenization.” How is it measured? Is it species-specific?

**Answer**. Thank you for your comment and inquiries. The concept of over-homogenization and their effect on boar sperm motility are now included.

Line 396: This step is more accurately described as “incubation with serotonin.” The term “homogenization of serotonin” is misleading.

**Answer**. Thank you for you comment. The text “At this point sperm homogenization is done but

serotonin homogenization is not; thus, homogenizations was performed at 5, 10, 15, 20 or 25

cycles.” was replaced with “At this point, sperm is homogenized, but serotonin incubation is still ongoing; hence, the sample was further mixed for 5, 10, 15, 20, or 25 cycles.”

Line 405: Note that 5 cycles were already shown in Experiment 1 to be sufficient for resuspension.

**Answer**. Thank you for your comment. Discussion was re-structured and this phrase was eliminated.

Line 414: Use “spermatozoa” instead of “sperm” when referring to individual cells.

**Answer**. Thank you for your comment. We have thoroughly reviewed the manuscript and revised the use of the terms “sperm” and “spermatozoa” to ensure consistency and accuracy when referring to individual cells.

Line 465: Why is an exact angle of rotation important in this context? Please justify.

**Answer**. Thank you for your inquiry. The angle of rotation is important because it simulates the natural forward and backward angular motion of the hand during manual homogenization. Therefore, ensuring repeatability of this movement is desirable to standardize the process and reduce operator-dependent variability. As stated in lines 130–134 of the original submission: “The movement performed by the tube holder is an inverted pendular motion at an 180-degree angle; we did choose that movement based on the hand movement for homogenization (See the video Hand_operated_homogenization.mp4 in the video section of <https://doi.org/10.17605/OSF.IO/ESFWG>).”

Line 468: The claim that timing is precise should be supported with data; where is this tested?

**Answer**. Thank you for you valuable comment and inquiry. The results of duration of cycles is in lines 293-301 of the original version of the manuscript. Raw data are available in data section at <https://doi.org/10.17605/OSF.IO/ESFWG>, specifically in <https://osf.io/sr96x/files/osfstorage>, please see the file Cycles_time.csv.

Line 479: “The use of CASHo in experimental settings highlights the importance of achieving

objective and repeatable homogenization without over-homogenizing.” : this was not tested nor discussed.

Answer. Thank you for you valuable comment. The sentence was rephrased as follow “The use of CASHo in experimental settings emphasizes the importance of identifying the point at which homogenization no longer alters sperm concentration, allowing the procedure to be stopped once an objective and repeatable state is reached.”

**Recommendation: Major Revision**
The manuscript has high potential and presents a technically useful contribution. However, substantial revisions are needed to clarify terminology, justify experimental design, and align the structure and interpretation with the core goal of developing and validating a reproducible, low-cost sperm mixer-homogenizer.

**Answer**. Thank you for your valuable comments. Each of them has been carefully addressed. We are confident that your review has helped us substantially improve the quality and clarity of our manuscript.
