# Supplementary material for: Development of an open-hardware semen homogenizer and application to serotonin effects on sperm motility
Source: PLoS One. 2025 Dec 5;20(12):e0338399. doi: 10.1371/journal.pone.0338399 (PMC12680138; doi:10.1371/journal.pone.0338399)
Supplement: Attachment — Submitted filename: Response_to_reviewers_auresp_2.docx [file pone.0338399.s007.docx]

The authors of the manuscript PONE-D-25-30420 “Welcome to the open-hardware sperm quality lab, featuring the new homogenizer: testing serotonin's effects on sperm motility” are grateful with Editor and reviewer's for their valuable time and suggestions to improve our manuscript. Each one of the comments and suggestions made by Editor and reviewers were addressed, as specified below. Please let me know any other comment or suggestion.

Best regards,

Dr. Andrés Aragón

Reviewer #1: The manuscript “Welcome to the open-hardware sperm quality lab, featuring the new homogenizer: testing serotonin's effects on sperm motility” is aims to design, construct, and validate an objective homogenizer for boar semen doses.

The revision substantially addresses the core reviewer concerns (design, modelling, structure, subpopulation analysis, clarity). The methodology is largely sound for a research article, with two moderate limitations: (i) absent morphology/functional markers and (ii) no empirical benchmarking vs. standard mixers. The statistical framework is appropriate overall, but I recommend (a) pairing/mixed modelling also at 5-cycle comparisons and (b) stating multiplicity handling or emphasizing effect sizes/CIs before acceptance.

I will recommend a moderate revision (address stats presentation at 5 cycles; add a brief multiplicity statement; explicitly flag morphology/functional markers as a limitation in Discussion; consider tightening the title). If these are implemented, I’d consider it publishable on the strength of its open-hardware contribution and transparent methodology

**Answer:** Thank you for your kind comments. We are grateful for your time and effort to help us to improve our manuscript. We hope that our responses and the corresponding modifications meet your expectations:

(i) We have added a statement in the Discussion explicitly acknowledging the absence of sperm morphology and integrity data as a limitation of the study. The text reads “Future research should investigate whether these factors influence sperm viability, membrane integrity, acrosome status, mitochondrial function, or DNA fragmentation, as their absence represents a limitation of the present study.”

(ii) We have added a statement in first paragraph of Discussion explicitly acknowledging the lacking of empirical benchmarking vs. standard mixers data. The text reads “CASHo represents a valuable alternative to commercial or open-hardware devices not specifically intended for semen homogenization. This assessment is based on technical information provided by manufacturers, as no direct experimental comparisons with commercial devices were performed in this study.”

(a) In the Statistics section, we originally wrote: “The effect of 5-HT at five cycles on total motility, progressive motility, and kinematic parameters (VCL, VAP, VSL, LIN, STR, WOB, BeatCross, and ALH) was compared using Student’s t-test,” because five cycles were considered the condition at which the sample was fully homogenized. Under this well-homogenized condition, we compared the Vh and 5-HT groups for total motility, progressive motility, and kinematic parameters. Subsequently, the five-cycle condition was used as the reference level in the mixed-effects model to analyze the over-homogenization conditions. To clarify we rephrased as follows “At five cycles, the effect of 5-HT treatment on total motility, progressive motility, and kinematic parameters (VCL, VAP, VSL, LIN, STR, WOB, BeatCross, and ALH) was assessed separately using a Student’s t-test, since this condition represents the point of complete homogenization and the comparison focused solely on treatment (5-HT vs. vehicle). Subsequently, the five-cycle condition was used as the reference level in the mixed-effects model, fitted to account for potential variability between boars, to evaluate over-homogenization effects across 10, 15, 20, and 25 cycles.”

(b) We thank the reviewer for this valuable suggestion. Because the aim of this study was exploratory rather than confirmatory, no formal multiplicity correction was applied. Instead, we placed emphasis on the magnitude and precision of the estimated effects, which are reported as effect sizes and 95% confidence intervals for fixed effects and interactions in S2 Table. This approach allows readers to evaluate the strength and direction of the observed effects beyond statistical significance.

Finally, the title was modified accordingly; it now reads “Development of an open-hardware semen homogenizer and application to serotonin effects on sperm motility.”

Reviewer #2: Manuscript title: Welcome to the open-hardware sperm quality lab, featuring the new homogenizer: testing serotonin's effects on sperm motility

Manuscript ID: PONE-D-25-30420_R1

Overall assessment

This manuscript presents the development and validation of an open-hardware semen homogenizer (CASHo) and its application in evaluating the effects of serotonin on boar sperm motility. The technical innovation is commendable and valuable for resource-limited laboratories. The operational definition of homogenization and the open availability of design files are important contributions.

However, the current manuscript in my opinion still requires major revisions. The main issues concern clarity of objectives, framing of the biological experiments, statistical transparency, and overemphasis on technical details. With revisions, this study could provide a meaningful resource for both bioengineering and reproductive biology.

Major comments

1. Clarity of objectives

The manuscript presents two parallel goals: (1) developing and validating the CASHo device, and (2) testing serotonin’s effects on sperm motility. At present, the serotonin experiment is insufficiently justified and feels appended.

Please clarify in the Introduction and Discussion whether the serotonin study is intended as a proof-of-concept demonstration of the device’s utility, or as a hypothesis-driven biological experiment. The framing will affect how results are interpreted.

**Answer:** Thank you for your valuable comment. We have clarified in the Introduction and Discussion that the serotonin experiment serves as a proof-of-concept demonstration of CASHo’s functionality, rather than a hypothesis-driven study. Corresponding sentences have been added to clearly frame the dual aims of the manuscript.

1.- In Introduction the objective 2 now reads: “As a proof-of-concept we use CASHo to investigate the effect of potential over-homogenization on the structure of kinematic subpopulations in sperm exposed to 5-HT in a non-capacitating medium.”

2.- In Discussion “In this study, the serotonin experiment was conceived as a functional demonstration of CASHo’s utility.”

2. Title and scope

The title (“Welcome to the open-hardware sperm quality lab…”) is catchy but misleading, since the work focuses on one device, not a full laboratory platform.

Consider a more precise title highlighting the homogenizer itself (e.g., “Development of an open-hardware semen homogenizer and application to serotonin effects on sperm motility”).

**Answer:** Thank you for your valuable comment. The title has been modified as suggested to: “Development of an open-hardware semen homogenizer and application to serotonin effects on sperm motility.”

3. Biological relevance and missing data

While motility subpopulation analysis is valuable, basic semen quality measures (total and progressive motility, morphology, integrity) should be reported alongside advanced kinematic parameters.

The absence of sperm morphology or integrity data limits assessment of the device’s impact on sperm quality. At minimum, please acknowledge this limitation more directly in the Discussion.

**Answer:** Thank you for your valuable comments. Total and progressive motility are presented in Figure 2. We have added a statement in the Discussion explicitly acknowledging the absence of sperm morphology and integrity data as a limitation of the study. The text readsThe text reads “Future research should investigate whether these factors influence sperm viability, membrane integrity, acrosome status, mitochondrial function, or DNA fragmentation, as their absence represents a limitation of the present study.”

4. Biological interpretation of clustering

While subpopulation analysis of sperm motility is an interesting approach, the manuscript does not fully explain what additional biological insights this provides compared to conventional mean values. For instance, the percentage shift (relative share) between subpopulations under different regimes and treatments should be visualized and discussed. This would clarify whether clustering reveals meaningful biological patterns (e.g., redistribution of sperm between more and less motile groups) beyond what is observed from average parameters.

At present, the added value of clustering remains unclear. Please expand the Discussion to explain what new information clustering contributes, how it should be interpreted biologically, and why it matters compared to traditional analyses.

**Answer:** Thank you for this insightful comment. Discussion was re-structured to highlight the advantage of subpopulation analysis over average analysis, mainly focused to the differential response of the kinematic subpopulation to 5-HT.

5. Comparisons with existing methods

The manuscript claims that commercial devices are unsuitable for gentle homogenization, but no direct experimental comparison is presented.

Please either:

a) provide direct comparative data (preferred), or

b) clearly state that claims are based on published studies and acknowledge the lack of head-to-head testing.

**Answer:** Thank you for your valuable comment. We have now clarified in the section “Limitations of existing commercial homogenization devices” that no direct experimental comparison with commercial equipment was performed. The statements regarding their limitations are explicitly based on previously published studies.

Specifically:

1.- The subheading was changed from “Effects of mixing using commercial devices” to “Limitations of existing commercial homogenization devices” to better reflect the scope of the discussion.

2.- The final paragraph of this section was rewritten to explicitly acknowledge that the discussion of commercial devices is drawn from prior reports and not from direct experimental testing.

3.- The following sentence was added at the end of the section:

“Although no direct comparison with commercial homogenizers was conducted, our discussion of existing devices and their reported effects is based on previously published studies. Therefore, claims regarding their limitations should be interpreted in this context, and future work should include direct comparative testing to validate these observations experimentally.”

These modifications ensure transparency about the evidence base supporting our statements and align the section with option (b) suggested by the reviewer.

6. Terminology and conceptual framing

The term homogenization is potentially misleading, as it usually refers to mechanical disruption of cells. Please clarify early in the text that the intended meaning is resuspension/mixing of spermatozoa and solutes.

Distinguish between different processes: (1) re-suspension of spermatozoa after sedimentation, (2) mixing of soluble additives such as serotonin, and (3) incubation for biological effects.

**Answer:** Thank you for your valuable comment and suggestions. The intended meaning of homogenization was clarified in Introduction, to read “Sample homogenization—mixing sufficiently to ensure consistent sperm cell counts is essential for semen evaluation, both in the production of insemination doses—to ensure consistent sperm cell counts—and in research applications, to obtain representative and reliable measurements.” In addition, the Material and methods section now explicitly distinguishes between three processes:

(1) resuspension of spermatozoa after sedimentation, now reads “During storage, sperm cells tend to sediment, making resuspension of spermatozoa is required prior to use.”

(2) mixing of soluble additives such as serotonin, now reads “...but incubation with 5-HT was still ongoing, requiring additional mixing of the additive.”

(3) incubation for biological effects, now reads “Sperm motility was evaluated immediately after each set of cycles.”

7. Over-homogenization

The concept of “over-homogenization” is central but underdeveloped. Please define it operationally (e.g., cycles beyond stabilization of sperm concentration) and explain its biological consequences.

Clarify whether this is species-specific (boar semen) or a generalizable phenomenon.

**Answer:** Thank you for your valuable suggestion.

1.- We have included the operational definition of over-homogenization in the subsection “Operational definition of objective homogenization and over-homogenization of semen samples.” The text now reads:

“A semen sample was considered over-homogenized when the number of mixing cycles applied in CASHo exceeded the level required to achieve homogenization.”

2.- The following clarification regarding the potential biological consequences of over-homogenization has been added to the Future directions:

“Because the number of cycles required to reach objective homogenization may vary across species, the threshold at which over-homogenization occurs is also likely to differ….Furthermore, studies across different species and extender formulations could clarify whether the impact of homogenization cycles and serotonergic signaling is generalizable or context-dependent.”

**Answer:** 8. Discussion writting

In several places, the Discussion begins with references to other studies rather than with the authors’ own findings. It is generally clearer to start each paragraph by restating the relevant result from the current study, and then relate it to existing literature. For example, in lines 557–565, the text does not clearly connect the study’s own data to the cited work. I recommend restructuring the Discussion so that the authors’ results are presented first, followed by comparison with previous findings (whether in agreement, contradiction, or expansion), along with possible explanations for similarities or differences. This will strengthen the logical flow and emphasize the study’s contribution.

**Answer:** Thank you for your valuable comment. The Discussion section has been revised and reorganized in several parts (including the section highlighted in your comment) to present our results first, followed by comparisons with previous studies. This modification improves the logical flow and emphasizes the contribution of the present study.

Minor comments

Organization and length

The revised structure (Methods following Introduction, supplementary hardware details moved to OSF) is much better. However, the main text is still too long (wordy). Consider further condensing hardware descriptions and focusing more on biological findings.

**Answer:** Thank you for your valuable suggestion. We have reviewed the hardware and operational descriptions once again and condensed them wherever possible. The remaining content was kept to the minimum necessary to ensure that the design and functioning of CASHo are comprehensible and reproducible. We believe this level of detail is essential for readers to fully understand the device and its validation.

1. Figures and tables

Some figures remain redundant or overly detailed. For example, raw individual variability plots may be better suited as Supplementary Materials, with main text showing summarized results.

In addition to reporting kinematic parameters, the manuscript could benefit from a visualization of how the relative proportion of sperm subpopulations shifts under different homogenization regimes and treatments. Presenting percentage shares (e.g., stacked bar plots, mosaic plots, or similar) would make these shifts more intuitive and highlight whether serotonin or over-homogenization leads to redistribution between motile and less motile clusters.

**Answer:** Thank you for your valuable comments.

1.- We are not entirely sure which specific figure you consider potentially redundant. Are you referring to Figure 4? This figure includes individual data points in panels B and C and, together with Table 2, presents the results of the principal component analysis, the selection of the optimal number of clusters, and the hierarchical clustering of averaged data. We consider Figure 4 essential for understanding the analytical workflow described in the Statistics subsection. Please let us know if we have correctly interpreted your suggestion.

2.- We agree that the mosaic plot (Figure 5) primarily illustrates statistical associations between treatment, number of cycles, and cluster membership, but does not directly convey the biological redistribution of sperm among kinematic subpopulations. To address this, we have now included complementary stacked bar plots (new Figure 5B and 5D) showing the relative proportions of each cluster under the different treatments and homogenization cycles. These visualizations make it clear that 5-HT exposure and over-homogenization induced a redistribution of sperm between clusters with distinct motility characteristics, even when average motility parameters remained unchanged. This addition clarifies the biological interpretation of the clustering analysis and highlights how subpopulation analysis reveals changes that would be masked by conventional mean-value comparisons.

2. Language and style

Although professionally edited, some vague terms remain. For example:

“Objective homogenization” → clarify with operational definition at first mention.

Replace observational wording (“we observed”) with more precise analytical phrasing where appropriate.

Ensure consistent use of spermatozoa (cells) versus semen (ejaculate fluid).

**Answer:** Thank you for your valuable comments. The following clarifications and actions have been implemented:

1. The term objective homogenization first appears in the Abstract (Line 32): “However, no objective or operational definition of homogenization—i.e., one that is quantifiable and repeatable—has been established.” The operational definition of objective homogenization is detailed in the subsection “Operational definition of objective homogenization of semen samples” within the Materials and Methods: “A semen sample was considered homogenized when sperm concentration in an aliquot did not significantly change after subjecting the sample to varying numbers of cycles in CASHo. A semen sample was considered over-homogenized when the number of mixing cycles applied in CASHo exceeded the level required to achieve homogenization.”

2. The wording “we observed” has been replaced with more analytical phrasing throughout the manuscript, as suggested.

3. The manuscript has been thoroughly revised to ensure consistent and appropriate use of the terms spermatozoa (cells) and semen (ejaculate fluid).

Recommendation

I recommend major revision. The technical contribution is strong and the study has real potential, but the biological framing, clarity of objectives, and statistical transparency must be improved before publication.

**Answer:** Thank you for your kind comments, time and your effort to help us to improve our manuscript. We have addressed all of you recommendations; we hope meet all your expectations.

Reviewer #3: I want to thank the authors for the work done. The revision addresses several concerns raised by the reviewers. Methods are clearer, the statistics read more smoothly, figures and tables are better organized, and the rationale for including serotonin (5-HT) has more substance than before. Thank you for the careful work. To help the biology stand alongside the engineering, and so that the paper reads as a full study rather than a device demonstration, adding a few reader-oriented clarifications would make a noticeable difference. Below are my major and minor comments:

The biological narrative is almost there but still feels under-explained in places. In the Results, it would help readers if the motility clusters were briefly characterized in biological terms and not only statistically. A small table or inset with centroids (mean ± SE for the standard CASA variables) and a short descriptive label for each cluster would anchor the interpretation. Alongside that, the text and figure captions would benefit from one or two plain sentences that say, in straightforward language, how VCL, VAP and VSL change across cycles in vehicle versus 5-HT, including any non-monotonic patterns. Naming the reference condition explicitly (for example, vehicle at 5 cycles) will keep the reader oriented. These are small additions, but they will make the biology feel complete and not merely illustrative.

**Answer:**  Thank you for your kind comments, time, and effort to help us to improve our manuscript. We have addressed all of you recommendations; we hope meet all your expectations:

1.- In the Results a short label for each cluster was added.

2.- Table with centroid would potentially duplicate the results, as editor noted in the first round of revision (where such as table was included).

3.- The Figure captions were modified to include the above suggestions.

4.- The level of reference is now explicitly named.

A related point concerns the captions themselves. Many readers, especially reading online (which is exactly the case of PLOS), arrive at the figures first and may rely on them heavily. It would be worth expanding the captions so they stand on their own: state what the plot shows, **what comparison or test is displayed**, the reference level used for contrasts, **and the sample size for each panel**. For the mosaic or heatmap-style figures, a single sentence on “how to read this” (e.g., what positive/negative residuals mean) will lower the barrier for speedy comprehension.

**Answer:** Thank you for your valuable comments and suggestions. All figure captions have been carefully revised and expanded to make the content more self-explanatory and accessible to readers. Each caption now specifies what the plot shows, the statistical tests and reference levels used, the sample size for each panel, and—where applicable—includes a brief note on how to interpret the mosaic or heatmap-style figures.

On the presentation of the device, a few “features” are currently placed in the Methods section as if they had been empirically established, although most are not demonstrated in the present study. Those statements would sit more comfortably in the device description as design intent or potential applicability, while the Results remain strictly limited to what was tested in boar semen under the reported conditions. This keeps the tone conservative without dampening the ambition of the tool (which was probably the intent of the authors, but not good for proper scientific paper).

**Answer:** Thank you for your valuable comment. The features of CASHo have been reworded and relocated to the beginning of the subsection “Hardware description.” The tone has been adjusted to emphasize the design intent, as suggested.

Title choice is, of course, the authors’ decision. From a reader’s perspective, the current wording still suggests that the serotonin component shares equal billing with the device. A slightly more device-forward title would set expectations cleanly, while the strengthened biological explanations above would preserve the balance inside the paper. If the title is retained, ensuring that the Results and Discussion deliver the clear biological takeaways sketched above should address the potential mismatch.

**Answer:** Thank you for your valuable comment. The title has been modified to emphasize the development of the device, now reading: “Development of an open-hardware semen homogenizer and its application to the study of serotonin effects on sperm motility.”

The transition in the Introduction from device motivation to the serotonin example remains a little abrupt. A neutral linking sentence around the lines 118-119 would resolve this. For instance: “Proper re-suspension of semen is particularly critical when evaluating the effects of exogenous agents on spermatozoa function (e.g., responses to serotonin), because uneven mixing can confound exposure and bias motility readouts.”

**Answer:** Thank you for your kind and valuable suggestion. We have followed your recommendation and added a neutral linking sentence based on your proposed wording to improve the transition between the device motivation and the serotonin example in the Introduction.

Language and terminology are much improved but a few edits remain (see the Line comments below). A light copy-edit will catch the residual duplications and errors. On statistics, the current description is clearer; if feasible, adding effect sizes or confidence intervals for the primary outcomes and a brief note that assumptions were checked would enhance transparency. One sentence that states the experimental unit for each analysis, and how biological versus technical replication was handled, would also help readers follow the modeling choices. Finally, if precision of angle or timing is presented as an empirical outcome rather than a design specification, pointing to the relevant QA will close that loop.

In sum, only modest refinements are needed: a clearer biological “voice” for the clusters and velocity patterns, fuller captions that work for web readers, and conservative placement of undemonstrated device features. With these in place, the manuscript will read as a balanced, full-value study that showcases the homogenizer and what it enables

**Answer:** Thank you for your valuable and constructive comments. We have carefully addressed all your recommendations in the revised manuscript. Language and terminology were refined through a thorough copy-edit; emphasis in confidence intervals, and clarification of experimental units and replication were made. Captions and brief descriptions of cluster patterns were revised to strengthen the biological interpretation, and statements regarding device performance were made more conservative. We hope these revisions meet your expectations and help present the manuscript as a balanced and complete study.

Line 107: “Given this lack of standardization, one consistent and fundamental aspect of homogenization is the manual movement of the tube.” - consider “remains the manual movement of the tube.”

**Answer:** Thank you for your suggestion. The sentence has been revised as recommended to read: “Given this lack of standardization, one consistent and fundamental aspect of homogenization remains the manual movement of the tube.”

Lines 114–115:“Despite this lack of standardization, one consistent and fundamental aspect is the manual movement of the tube.” - duplicates Line 107; please remove.

**Answer:** Thank you for noting this duplication. The repeated sentence has been removed from Lines 114–115.

Line 116: “The design…” (capital “T” appears lost).

**Answer:** Thank you for noting “T” absence. The “T” was added.

Line 133: “additional homogenisation” - this is a good place to introduce and define “over-homogenization”; you use the term later in the aims.

**Answer:** Thank you for your valuable suggestion. We have incorporated the following clarification into the text:

“We refer to this additional homogenization step as over-homogenization, defined as the condition in which a semen sample is mixed beyond the level required for standard homogenization.” An operational definition of over-homogenization is in the subsection

Line 167: “device” (minor typo).

**Answer:** Thank you for noting this typo. The word “devise” was corrected.

Lines 283–284: Please rephrase for clarity: “At this stage, the semen samples were homogenized, but incubation with 5-HT was still ongoing. The samples were then mixed for 5, 10, 15, 20, or 25 cycles.”

**Answer:** Thank you for your helpful comment. The sentence has been rephrased for clarity following your suggestion, while ensuring that the revised text accurately reflects the sequence and purpose of the procedures:

“At this stage, the semen samples were homogenized, but incubation with 5-HT was still ongoing. The control samples were then mixed for 5 cycles; other samples were mixed in conditions of over-homogenization during 10, 15, 20, or 25 cycles.”

Line 375: “After 5 to 25 cycles, the sperm concentration stabilized, reaching …” - clarify after how many cycles stabilization occurs (e.g., “stabilized by 5 cycles”).

**Answer:** Thank you for your comment. The text was modified as follow “Sperm concentration stabilized after five cycles and remained at a plateau through 25 cycles in both semen stored for less than 5 hours and semen stored for 24 hours (Fig. 1).”

Line 395: “five cycles at 4.9 ± 2.9 seconds with an inclination angle of 180 degrees” - it is unclear how the cycle duration was chosen; please state the criterion/rationale.

**Answer:** Thank you for your valuable comment. The inclination angle and cycle duration were determined empirically. We recorded and analyzed videos of manual homogenization performed under what we considered gentle mixing conditions to estimate both the angular displacement and the average duration of a complete cycle. This information was originally presented in Lines 185–188 of the previous version of the manuscript: “A video (Hand_homogenization.mp4, available in the Videos section at https://doi.org/10.17605/OSF.IO/ESFWG) demonstrates the proper hand positioning for holding a tube containing a semen sample, along with the angular forward and backward movements used during manual homogenization.”

We have revised the text to explicitly state that the rationale for selecting the 180° inclination and the 4.9 ± 2.9 s cycle duration was based on these empirical observations of manual mixing behavior. The following text was included at the begin of section Validation of CASHo: “The inclination angle (180°) and cycle duration (4.9 ± 2.9 s) were empirically determined by recording and analyzing videos of manual homogenization performed under gentle mixing conditions. These recordings (see the video example Hand_homogenization.mp4 available in the Videos section at https://doi.org/10.17605/OSF.IO/ESFWG) were used to estimate the average angular displacement and the time required to complete one forward–backward movement of the tube. These empirical observations provided the rationale for defining the standard CASHo cycle.

Line 396: “motility. At this stage, the semen sample was homogenized, but incubation with serotonin was still ongoing.” - please add where/when 5-HT was added relative to baseline mixing and subsequent cycles.

**Answer:** Thank you for your helpful comment. We have now clarified the timing of 5-HT addition relative to the homogenization process: “After the semen sample was homogenized for five cycles, 5-HT was added, and the sample was subsequently homogenized again for 5, 10, 15, 20, or 25 cycles. The treatment with five cycles served as the reference condition; whereas the additional cycles (10–25) represented over-homogenization, intended to evaluate whether excessive mechanical mixing during incubation affects sperm motility or the response to 5-HT.”

Line 415: “Sperm exposed to 5-HT exhibited reduced total and progressive motility” - compared to what?

**Answer:** Thank you for your valuable inquiry. The following text was added to clarify “Sperm exposed to 5-HT exhibited reduced total and progressive motility at 25 cycles compared with control at same number of cycles (Fig 2)”.

Line 416: “Neither the effect of cycles nor the interaction between treatment and cycles was significant” - in which group/analysis, please specify.

**Answer:** Thank you for your valuable comment. The text was modified to clarify: “According to the ANOVA results, neither the effect of cycles nor the interaction between treatment (5-HT vs. vehicle) and cycles was significant for total or progressive motility.”

Line 417: “However, significant effects of 5-HT, cycles, and their interaction were observed” - for which comparisons (e.g., at >15 or 25 cycles?)-please specify.

**Answer:** Thank you for your valuable comment. The effects of 5-HT, cycles, and their interaction (cycles × treatment) varied among the different motility parameters. Statistical results for each parameter at every level of treatment, cycle, and interaction are detailed in Supplementary Table S2. We decided to include these data as supplementary material to avoid excessive length in the main text. However, in Figure 3A, these effects can be visually appreciated: when the standard error bars overlap, there is no significant difference between treatments; when the lines do not overlap, a significant difference is present; and when the treatment lines cross, this indicates a significant cycles × treatment interaction. In this sense, Figure 3B displays the lowest p-value obtained for each kinematic parameter for treatment, cycles, and their interaction. We also added the text “Individual p-values for the effects of treatment, cycles, and their interaction (cycles × treatment) are provided in S2 Table.”

Line 495: “Sperm in Cluster 3 were less responsive to 5-HT treatment but remained sensitive to …” - do you mean no significant 5-HT effect at the initial step before additional cycles? Please clarify the intended comparison.

**Answer:** Thank you for your comment. We have modified the sentence as follows:

“In Cluster 3, no significant effects of 5-HT were detected at 5, 20, and 25 cycles, although WOB and BeatCross remained sensitive to the effects of cycles and to the cycles × 5-HT interaction.”

In addition, we added the following text: “Individual p-values for the effects of treatment, cycles, and their interaction (cycles × treatment) are provided in S2 Table.”

Line 506 - “Capabilities and limitations” - some items were tested here, others not; please separate demonstrated vs prospective capabilities (or cite prior validation if applicable).

**Answer:** Thank you for your valuable comments. The text has been revised to explicitly distinguish between tested and prospective capabilities within the “Capabilities and Limitations” section.

Line 513 - “CASHo enables objective studies on semen homogenization.” - too general; consider a more concrete wording tied to the operational definition and reproducible cycles.

**Answer:** Thank you for your valuable comment. The sentence has been revised to read: CASHo enables objective and reproducible studies on semen homogenization by allowing precise control of rotation speed, angle, and number of cycles.

Lines 514–515 - “The 50 mL tube holder can be replaced with holders of other capacities (e.g., 2 mL or 15 mL).” - Were these tested? If not, frame as potential applicability; if yes, state evidence that mixing is adequate in smaller tubes.

**Answer:** We appreciate this helpful comment. The sentence “The 50 mL tube holder can be replaced with holders of other capacities (e.g., 2 mL or 15 mL)” has been revised to present this feature as a prospective capability.

Lines 529-530: “commercial equipment cannot be easily …” - consider “existing commercial equipment tested in prior reports cannot be easily …” (and good to cite the specific sources).

**Answer:** Thank you for your recommendation. The sentence has been rephrased according to your suggested wording: “existing commercial equipment tested in prior reports cannot be easily …”, and the relevant sources have been cited.

Line 586:“thus, long-term semen extenders” - consider “extenders for long-term storage.”

**Answer:**  Thank you for your suggestion. The text was modified accordingly.
