# Supplementary material for: Development of an open-hardware semen homogenizer and application to serotonin effects on sperm motility
Source: PLoS One. 2025 Dec 5;20(12):e0338399. doi: 10.1371/journal.pone.0338399 (PMC12680138; doi:10.1371/journal.pone.0338399)
Supplement: Attachment — Submitted filename: PONE-D-25-30420_R3-AE Comments.docx [file pone.0338399.s008.docx]

Development of an open-hardware semen homogenizer and application to serotonin effects on sperm motility

Nancy Juárez-Contreras ^1^, Cindy U. Rivas-Arzaluz ^1#^, Oscar Gutiérrez Pérez^2^, María E. Ayala^3^, Andrés Aragón-Martínez^1,^*

^1^Laboratorio de Gametos y Desarrollo Tecnológico, Unidad de Biomedicina, Facultad de Estudios Superiores Iztacala, UNAM, Estado de México, Mexico, CP 54090.

^2^Centro de Enseñanza, Investigación y Extensión en Producción Porcina (CEIEPP), FMVZ -UNAM, Estado de México, Mexico, CP 54240

^3^Unidad de Biología de la Reproducción, Laboratorio de Pubertad, Facultad de Estudios Superiores Zaragoza, UNAM, Ciudad de México, Mexico, CP 15000.

^#^Current Address: Departamento de Genética del Desarrollo y Fisiología Molecular, Instituto de Biotecnología, UNAM, Cuernavaca, Morelos, México, CP 62210.

*Corresponding author

Email: [andres.aragon@iztacala.unam.mx](mailto:andres.aragon@iztacala.unam.mx)

Short title: An open-hardware semen homogenizer

# Abstract

Proper homogenization of semen samples is crucial for assisted reproductive technology (ART) and analysis of sperm motility kinematics in experimental research. Accurate homogenization ensures a consistent number of sperm per insemination in ART and uniforms number of sperm per treatment in research. Homogenization is particularly important when sperm are treated with exogenous compounds that must be evenly distributed throughout the experiment. Traditionally, semen samples are homogenized manually with a “gentle” approach or with commercial devices that are not specifically designed for this purpose. However, no objective or operational definition of homogenization—i.e., one that is quantifiable and repeatable—has been established. In this study, we designed, constructed, and validated an open-hardware semen homogenizer for sperm motility analysis. For validity, the hardware was used to investigate the effects of serotonin (5-HT) on boar sperm kinematics at the subpopulation level using a computer-assisted sperm analysis (CASA). All components of the homogenizer are readily available, including the STL files for the 3D-printed parts, firmware, assembly instructions, and operation guidelines. Objective homogenization was evaluated by measuring sperm density at different numbers of homogenization cycle, while “gentle” homogenization was assessed based on manual duration and inclination angle. The homogenizer provided precise control over cycle duration and rotation angle. Sperm motility kinematics showed no changes at the average level, however distinct effects emerged within subpopulations. Exposure to 5-HT differentially affected sperm motility kinematics across subpopulations displaying no change, increase or decrease in velocity indices. Under over-homogenization conditions, a clear interaction between prolonged homogenization and 5-HT exposure was observed in all subpopulations. Results suggest that objective homogenization is essential to ensure consistent sperm density ~~and reliable exposure to 5-HT~~, whereas over-homogenization alters sperm responses, potentially leading to biological misinterpretations.

## Keywords

CASA; Homogenization of semen; Sperm density; Sperm motility kinematics; Serotonin

# Introduction

In our lab semen quality is routinely evaluated with aid of open-hardware. In this context, glass slides and plastics are heated on a thermal plate constructed with the hot bed of a 3D printer, sperm motility kinematics are analysed using a an open-source computer-assisted sperm analysis system (CASA) [1], and sperm density and vertical velocity are evaluated in the UPSPERM device [2]. Semen sample homogenization—mixing sufficiently to ensure consistent sperm cell counts is essential for semen evaluation, both in the production of insemination doses—to ensure consistent sperm cell counts—and in research applications, to obtain representative and reliable measurements. Homogenization of semen ensures consistent sperm density required for operation of fertilization test or CASA. Obtaining a representative sperm sample is fundamental for statistical analysis, as it ensures that all sperm—regardless of their cellular or molecular characteristics—have an equal probability of being selected for measurement.

In most laboratories, homogenization of semen is typically performed in a tube by means of manual operation using repeated forward and backward rotational movements (See the video Hand_homogenization.mp4 in videos section of [*https://doi.org/10.17605/OSF.IO/ESFWG*](https://doi.org/10.17605/OSF.IO/ESFWG))~~, until suspension appear uniform by eye~~. This process is often described as “gentle homogenization” in scientific reports [3,4]. Gentle homogenization is necessary because vigorous agitation may cause damage to the sperm cells or collisions between sperm and the tube walls~~, and exposing the aqueous interface to air~~ [5–8]. In practice, gentle homogenization is often understood as handling the sample slowly; however, “slowly” remains a subjective term. Human factors affect semen homogenization as different technicians may perform varying numbers of rotational movements at different speeds that may potentially cause damage to the sperm cells.

A key application of semen homogenization lies in the storage and handling of semen samples. Among the commercial devices used to “blend/mix/agitate/rock” samples are orbital mixers/shakers, rotational mixers, nutating mixers, reciprocating mixers, and hand-motion shakers (https://www.coleparmer.com/c/shakers). These proprietary devices are multi-purpose and not specifically designed for semen homogenization, but they can be adapted to simulate hand movements. For example, the Multi Bio RS-24 (https://www.boeco.com/mixer-stirrer-and-rotator/boeco-rotator-multi-bio-rs-24&sk=78, approximately 675 dollars before VAT in México) is described as a programmable rotary agitator, which allows adjustment of rotation speed, angle, reciprocal rotation, and time interval. However, the number of inversions and recoils must still be determined by eye and experimentation. Another option potentially adaptable to gentle homogenization is the Cole-Parmer WA-200 Hand Motion Flask Shaker (https://www.coleparmer.com/i/cole-parmer-wa-200-hand-motion-flask-shaker-230-v/5190002) (2550.00 dollars at October 2024). This device, however, has a restricted rotation angle and high velocity (80 to 800 RPM). According to the manufacturer, the hand Motion Flask Shaker is intended for prolonged and continuous shaking, making it less suitable for handling sensitive biological samples such as semen.

To avoid sampling bias, the World Health Organization (WHO) recommends mixing semen before taking aliquots:

Before removing an aliquot of semen for any assessment, mix the sample well in the original container, but not so vigorously that air bubbles are created. Mixing can be achieved by placing the specimen container on a moving tray during liquefaction in a 37 °C incubator. In the absence of an orbital mixer, a basic mixing could be achieved by approximately 15–30 seconds of manual swirling [9].

A portable open-hardware laboratory rotator mixer and shaker designed for low-capacity tubes, arranged in a mirrored configuration, has been reported previously [10]. Two hardware versions exist: The laboratory rotator mixer and shaker exist in two forms: the first provides continuous 360° rotation while lacking velocity control, and the second uses a programmable microcontroller to regulate the rotation angle with a limited inclination range. These devices are intended primarily for mixing chemical solutions, not for handling sensitive biological samples such as semen. Moreover, both versions rely on DC motors, which require additional electronic components to regulate velocity and angular movement with precision.

Given this lack of standardization, one consistent and fundamental aspect of homogenization remains the manual movement of the tube. We define full homogenization of the sperm suspension as the point at which no further changes in sperm concentration are observed after the homogenization procedure has been performed. Homogenization of semen samples typically involves rotational forward and backwards motion, however duration of motion may affect the results, the inclination angle remains undefined, and no criteria exist for determining when the process should stop. In practice, homogenization is usually concluded based on visual inspection or after an arbitrary period.

The design and construction of an open-hardware device specifically intended for semen homogenization represents a feasible alternative to modifying commercial equipment or adapting open-hardware mixers and shakers that were not originally designed for semen homogenization. Proper re-suspension of semen is particularly critical when evaluating the effects of exogenous agents on sperm function ~~(e.g., responses to serotonin)~~, because uneven mixing can confound exposure and bias motility readouts. For the case of serotonin (5-HT), sperm has been reported to express proteins related to serotonergic communication, including receptors, transporters, and metabolizing enzymes [11]. As an example, an increase in sperm velocity has been reported when human non-capacitated sperm was treated with 5-HT. Other studies have reported changes in average motility parameters of hyperactivated hamster sperm exposed to 5-HT receptor agonists or antagonists [12,13]. Despite these findings, the structure of kinematic subpopulations of sperm exposed to 5-HT remains unknown. A novel computational approach previously identified alterations in the kinematic subpopulation structure of boar sperm exposed to ketanserin (an 5-HT₂ receptor inhibitor), with results showing increased velocity accompanied by reduced linearity [14]. None of these studies addressed the problem of semen sample homogenization.

The procedure for exposing sperm to a substance such as 5-HT requires that a previously homogenized semen sample be taken from a container. Once the substance is added, the semen is homogenized but the substance is not; therefore, the sample must be homogenized again to ensure uniform exposure. We refer to this additional homogenization step as over-homogenization, defined as the condition in which a semen sample is mixed beyond the level required for standard homogenization. Whether this additional homogenization step affects kinematic outcomes remains unknown.

The objectives of this study were (1) to design and construct an open-hardware device capable of inverting and recoiling at defined inclination angles and velocities, and repeating the procedure a predetermined number of times. This device was named the “Customizable Automated Semen Homogenizer” (CASHo). (2) As a proof-of-concept we use CASHo to investigate the effect of potential over-homogenization on the structure of kinematic subpopulations in sperm exposed to 5-HT in a non-capacitating medium.

# Material and methods

## Hardware description and operation instructions

The CASHo was designed to provide homogeneous mixing and reduce operator variability during the handling of cell suspensions. The system features a fully customizable structure, encompassing both the aluminum framework and the firmware, allowing users to adjust the device according to specific laboratory requirements. Its tube holder was conceived to accommodate tubes of different capacities, ensuring versatility across experimental setups. In addition, the rotation speed, angle, and number of cycles can be configured through the firmware, offering precise control over the mixing process. The design is also scalable, permitting expansion of the tube holder to process a higher number of samples when needed. Although developed primarily for semen homogenization, the CASHo was intended to enable objective mixing of other types of cellular suspensions where reproducible mechanical agitation is desirable.

CASHo consists of an extruded aluminum base and a set of 3D-printed components that support a stepper motor and custom-made parts designed to hold a 50 mL tube. The electronic control board is an Arduino UNO, which receives instructions via Arduino App ([https://www.arduino.cc](https://www.arduino.cc/)) through a computer serial monitor. The tube holder performs an inverted pendular motion at a 180-degree angle, a movement selected because it replicates the hand motion during manual homogenization (see the video Hand_homogenization.mp4 in the Videos section of [*https://doi.org/10.17605/OSF.IO/ESFWG*](https://doi.org/10.17605/OSF.IO/ESFWG)).

Commercial multipurpose devices available on the market can regulate movement speed but do not reproduce the motion typically performed by technicians during homogenization. By contrast, CASHo ~~has been specifically designed for semen sample homogenization,~~ accounts for both speed and motion of hand-operated techniques. The device enables the application of objective definitions of homogenization and “gentle homogenization.”

CASHo ~~is an affordable device suitable for laboratories with limited resources. Its~~ cost represents approximately 22% of the least expensive commercial option and 6% of the most expensive device currently available. All components are low-cost and readily accessible through e-commerce platforms. A detailed list of components, with reference links, prices and suppliers, is provided in S1 Table (Supplementary Information, [*https://doi.org/10.17605/OSF.IO/ESFWG*](https://doi.org/10.17605/OSF.IO/ESFWG)). Unlike commercial devices, which generally allow adjustments of speed and angle but not a defined number of cyclic movements, CASHo enables precise modification of the number of cycles, rotation angle, and speed through firmware adjustment. This flexibility allows adaptation to semen homogenization across different species. The devise may also be adapted for homogenizing other cell suspensions, although technical parameter adjustments and validation of effectiveness are required on a case-by-case basis.

Several components of CASHo were designed for 3D printing using OpenSCAD ([https://openscad.org](https://openscad.org/)) (Table 1). The design files (STL), code source (scad), and supporting information are in the STL section at https://doi.org/10.17605/OSF.IO/ESFWG; whereas details on design files, components and mounting details are in supplementary material (AAAAAAA) the file CASHo _Supplementary_Details.pdf in the Supplementary section at <https://doi.org/10.17605/OSF.IO/ESFWG> .

| **Table 1. Design files of the 3D-printed parts of CASHo**. | | |
| --- | --- | --- |
| **Design file name** | **File type** | **Open source license** |
| **Junction_piece** | STL, scad | GNU GPLv3 |
| **Rod_adapter** | STL, scad | GNU GPLv3 |
| **Tube_holder** | STL, scad | GNU GPLv3 |
| **Nema_support** | STL, scad | GNU GPLv3 |
| **Rod_support** | STL, scad | GNU GPLv3 |
| **Hand_operated_homogenization** | mp4 | GNU GPLv3 |
| **Device_homogenizing_process** | mp4 | GNU GPLv3 |
| **Agitador_v6** | ino | GNU GPLv3 |

A video (Hand_homogenization.mp4, available in the Videos section at <https://doi.org/10.17605/OSF.IO/ESFWG>) demonstrates the proper hand positioning for holding a tube containing semen sample, along with the angular forward and backward movements used during manual homogenization. Another video (CASHo_homogenization.mp4, available in the Videos section at <https://doi.org/10.17605/OSF.IO/ESFWG>) presents the fully assembled CASHo device connected to a computer via USB. The video shows the Arduino serial monitor with instructions for initiating a work routine, as well as real-time output displaying the number of cycles and the duration of each cycle. The firmware file Homogenizador_v6.ino (available at <https://doi.org/10.17605/OSF.IO/ESFWG>) is used to control the device; it includes variable definitions and specifies the pin configuration required to interface with the stepper motor driver.

Detailed operating instructions for CASHo are available on protocosl.io (<https://dx.doi.org/10.17504/protocols.io.4r3l29ew3v1y/v1>). ~~A shortened version is provided below.~~

### Pre-Safety Checks

- Ensure that the aluminum base and all parts are securely fastened.
- Confirm that all wiring is properly connected and tightly secured.
- Verify that the power supply is switched off.

Launch the Arduino IDE on the computer, open the file Homogenizer_v6.ino, and verify communication between the IDE and the Arduino UNO board. Uploading the firmware is required only once, unless modifications are made to the code in Homogenizer_v6.ino.

Ensure that the Tube_holder is in vertical position, which represents the original starting point.

Switch on the power supply to activate the stepper motor driver. Hold the Tube_holder to confirm that it remains stable, and insert a tube containing diluted semen.

Open the serial window in the Arduino IDE and verify that communication is established at 9600 baud. Enter “1” or “2” in the input section of the serial window to begin the routine. The motor shaft will then rotate, producing movement of the Tube_holder.

The output section of the serial monitor window displays the number of cycles defined in the routine, as well as the duration of each cycle in seconds (see CASHo_homogenization.mp4 at <https://doi.org/10.17605/OSF.IO/ESFWG>).

## ~~Operational definition of objective~~ Homogenization and over-homogenization of semen ~~samples~~

Currently, no objective criterion exists to determine when a semen sample is well homogenized. The initial step consisted of determining, with CASHo, the number of cycles required to achieve homogenization in fresh semen (within five hours of collection) and refrigerated semen (after 24 hours of storage). A semen sample was considered homogenized when sperm concentration in an aliquot did not significantly change after subjecting the sample to varying numbers of cycles in CASHo. A semen sample was considered over-homogenized when the number of mixing cycles applied in CASHo exceeded the level required to achieve homogenization.

## The boars and semen collection

Diluted semen doses for artificial insemination were prepared at the Centro de Enseñanza, Investigación y Extensión en Producción Porcina (CEIEEP), Facultad de Medicina Veterinaria y Zootecnia (UNAM), and subsequently donated to the laboratory. Ejaculates were collected at CEIEEP using the gloved-hand technique, and semen doses were prepared with Androstar Plus extender (Minitube, Tiefenbach, Germany). The samples were transported under refrigeration for approximately three hours from CEIEEP to the laboratory.

~~Upon arrival at the laboratory,~~ Semen samples were (1) subjected to experimental procedures on the same day (within five hours of collection) or (2) used after 24 hours of storage. In the latter case, samples were stored refrigerated at 17°C in tubes placed horizontally.

Semen samples were obtained from five boars. Commercial semen extenders allow the preservation of boar semen for several days [4]; therefore, samples were stored at 17°C [1,5,6], as this temperature has been reported to provide slight benefits for the metabolic activity and membrane integrity of boar sperm [15]. During storage, sperm cells tend to sediment, making re-suspension of sperm is required prior to use. Examples of diluted boar semen stored in tubes placed vertically or horizontally for 24 hours are provided in the Figures section at <https://doi.org/10.17605/OSF.IO/ESFWG>.

The donor boars at CEIEEP were selected based on proven fertility and are routinely used in genetic dissemination programs for regional pig producers, as well as for pig production at the center’s experimental farm. A strict management protocol is followed, including weekly semen collections to ensure consistent semen quality in terms of volume and concentration. Collections with progressive motility (evaluated subjectively) below 80% or with more than 15% morphological abnormalities are routinely discarded. ~~Only ejaculates meeting these quality criteria were used in the present experiment.~~

~~The genotypes of the boars included Pietrain (n = 3), Yorkshire × Pietrain (n = 1), and Yorkshire × Landrace (n = 1).~~ The animals ranged in age from 1.8 to 2.3 years. ~~Semen samples were transported to the laboratory at 17 °C in accordance with commercial distribution protocols.~~

## Assessment of sperm concentration

Five samples, one of each of five boars were split in six aliquots. Each aliquot was subjected to 0, 1, 2, 3, 4, or 5 routines of five cycles each, corresponding to 0, 5, 10, 15, 20, or 25 total cycles. After each set of cycles performed with the CASHo device, sperm concentration was evaluated. Thus, each boar served as a block, and the experimental unit was the aliquot within boar. A 100 µL aliquot was collected with a micropipette by immersing the pipette tip halfway into the 50 mL tube. Each aliquot was placed at the bottom of a spectrophotometer cuvette (2.5 mL of capacity) containing 800 µL of Androstar Plus extender. At this stage, a heterogeneous sperm suspension was generated; homogenization within the cuvette was achieved by pipetting the full volume three times, with the beveled tip positioned at the bottom of the cuvette. Absorbance was then measured at seven distinct heights within the cuvette using the UPSPERM device, and sperm concentration was calculated [2]. Diluted semen samples were homogenized within the cuvette to enable accurate absorbance measurement [2]. Semen samples were then stored at 17°C for 24 hours, after which the same procedure of homogenization and sperm concentration evaluation was repeated. Individual measurements of sperm concentration were recorded as a function of time, boar ID, and number of cycles.

## The 5-HT effects on sperm motility kinematics after distinct number of cycles

A treatment group with zero homogenization cycles was not included in the experimental design. This decision was based on standard practices in semen analysis and handling. Ejaculates were analyzed within five hours of collection, and in semen processing, even minimal manipulation (e.g., gentle swirling or low-speed stirring) constitutes a form of mechanical mixing. Therefore, a true “no homogenization” control would not have been operationally feasible or biologically meaningful. Instead, we defined five homogenization cycles were defined as the minimum baseline, representing the lowest consistent and controlled level of mechanical input required to simulate routine sample preparation. This ensured that all samples met baseline handling standards while allowing evaluation of the degree of homogenization rather than its complete absence.

For the 5-HT exposure experiment, ten aliquots of 1000 µL each were taken from each of five semen samples: five served as control (Vh group) and five were treated with 5-HT at a final concentration of 10 µM. Each boar provided one biological replicate for each treatment, and aliquots within boar were treated as technical replicates. At this stage, the semen samples were homogenized, but incubation with 5-HT was still ongoing, requiring additional mixing of the additive. The control samples were then mixed for 5 cycles; other samples were mixed in conditions of over-homogenization during 10, 15, 20, or 25 cycles. Sperm motility was evaluated immediately after each set of cycles.

## Assessment of sperm motility

Sperm motility was evaluated as previously stated [1]. Briefly, 15 µl of sperm suspension was placed on a slide, covered with a clean 22 × 22 mm coverslip, and examined using a B3 Clinilab phase-contrast microscope (Motic, British Columbia, Canada) equipped with a customized stage prewarmed to 38 °C. All glassware and plastic materials were prewarmed to 38 ºC on an open-hardware heat plate.

For each sample, three to four image sequences from separate fields at 100× magnification (approximately 200 sperm per sample) were acquired using a Stingray F-033B camera (Allied Vision Technologies Inc., Exton, PA, USA) and stored digitally. Image sequences were captured with µManager software version 1.4 [16] at 60 frames per second (60 Hz) for two seconds. Image sequences were analyzed using ImageJ software version 1.50d [17] and the CASA plugin [1]. Sperm average path velocity (VAP, µm/sec), curvilinear velocity (VCL, µm/sec), straight-line velocity (VSL, µm/sec), beat cross frequency (BCF, Hz), linearity (LIN, VSL/VCL), straightness coefficient (STR, VSL/VAP), amplitude of lateral head displacement (ALH, µm), and wobble (WOB, VAP/VCL) were analysed. Kinematic data for individual sperm were used to construct the dataset, which is available on the OSF platform (<https://doi.org/10.17605/OSF.IO/ESFWG>).

## Statistical analysis

Assumptions such as normality of the data for all variables measured in this study were checked by the Shapiro–Wilk test and residuals. Mean of sperm concentration at each time point was compared with values at 0 or 5 cycles using one-way ANOVA followed by Dunnett’s test. Statistical significance was set at P<0.05. At five cycles, 5-HT effects on sperm motility kinematics was assessed separately using a Student’s t-test~~, since this condition represents the point of complete homogenization and the comparison focused solely on treatment (5-HT vs. vehicle)~~. Subsequently, the five-cycle condition was used as the reference level in the mixed-effects model, fitted to account for potential variability between boars, to evaluate over-homogenization effects across 10, 15, 20, and 25 cycles. The model was defined as

$$Y_{ijk}=\beta_{0}+\beta_{1}\cdot\left( Treatment \right)_{j}+\beta_{2}\cdot\left( Cycles \right)_{k}+\beta_{3}\cdot\left( \text{Treatment}\cdot Cycles \right)_{jk}+\upsilon_{i}+\epsilon_{ijk}$$

where Y*ijk:* Is the observed value of a kinematic variable (e.g., VCL, VSL) for the *i*-th boar, under treatment *j* and homogenization cycle *k*. $\beta_{0}$: Intercept. Estimated mean response for the vehicle group at 5 cycles (reference level). $\beta_{1}$: Main effect of treatment. Difference between serotonin and vehicle across all cycles. $\beta_{2}$: Main effect of homogenization cycles; effect of increasing the number of homogenization cycles (e.g., 5, 10, 15, etc.). $\beta_{3}$: Interaction effect; how the effect of serotonin changes depending on the number of homogenization cycles. $\upsilon_{i}$: Random effect for boar., capturing biological variability between boars (Boar_ID). $\epsilon_{ijk}$ ~ N(0,σ^2^): Residual error, representing variability within boars and within treatment-cycle combinations.

To identify sensitivity of kinematic subpopulations to 5-HT and to the number of homogenization cycles, the method described in [14] and the protocol for identification of kinematic subpopulations (<https://dx.doi.org/10.17504/protocols.io.6qpvr9e6zvmk/v1>) were followed. Briefly, individual values of motility kinematics were used as input for principal component analysis (PCA). Principal components (PC) with eigenvalues greater than one were then used as input for hierarchical clustering analysis. The number of clusters (subpopulations) was determined based on inertia gain (total variance gain).

The same mixed-effects model used to account for potential variability between boars was applied to motility parameters within each cluster. Significant main effects of treatment (Vh or 5-HT), number of homogenization cycles (10, 15, 20, or 25), and their interaction were obtained. A threshold of *P* < 0.05 was considered significant. Interactions between variables were visually inspected using interaction plots. Results are presented as mean ± standard error of motility parameters within each subpopulation, and P-values for the effects of treatment, number of cycles, and their interaction are presented as heatmaps.

Pearson’s chi-square test was applied to assess whether the distribution of sperm across kinematic clusters differed significantly between treatments. For this analysis, a two-way contingency table was constructed with Treatment and Cluster as categorical variables. To evaluate the joint distribution of sperm across clusters, treatment conditions, and cycle stages, a hierarchical mosaic plot was generated. This visualization was based on a log-linear model assuming complete mutual independence among the three categorical variables. Shading of the mosaic tiles reflected standardized Pearson residuals, highlighting deviations between observed and expected frequencies under the independence model [18]. A spacing argument was incorporated to improve interpretability by visually separating the levels of each factor. All statistical analyses were performed using R software version 3.4.4 [19] on a MacBook running macOS version 15.0.1.

# Results

## Validation of CASHo

The inclination angle and cycle duration were empirically determined by recording and analyzing videos of manual homogenization performed under gentle mixing conditions. These recordings (see the video example Hand_homogenization.mp4 available in the Videos section at https://doi.org/10.17605/OSF.IO/ESFWG) were used to estimate the average angular displacement and the time required to complete one forward–backward movement of the tube. These empirical observations provided the rationale for defining the standard CASHo cycle.

### Verification of angle of rotation

A clock face was printed on paper and affixed to the Nema_support using common glue. A 3D-printed ring with an arrow was attached to the flexible aluminium coupling, with the arrow positioned at 12:00 on the clock face. During operation, the angular movement of the arrow was visually verified to align with the clock face at 12:00 (original position), 03:00 (initial position), and 09:00. A demonstration of this procedure is provided in CASHo_homogenization.mp4 (Videos section, <https://doi.org/10.17605/OSF.IO/ESFWG>).

### Verification of velocity

Functionality of CASHo was verified by analyzing the duration of cycles and routines. The firmware logic is available in the Supplementary Information section (<https://doi.org/10.17605/OSF.IO/ESFWG>). A 50 mL tube containing 45 mL of diluted semen was placed in the Tube_holder; these samples were used exclusively for verification and not for subsequent experiments. Cycle durations were recorded directly from the serial monitor output, using 25 consecutive cycles to exceed the number typically required for manual homogenization. The mean cycle duration was 4909 ± 0.2949 milliseconds, with a coefficient of variation of 0.0060. The dataset is provided in the file Cycles_time.csv on the OSF platform (Data section, <https://doi.org/10.17605/OSF.IO/ESFWG>).

### Effect of cycles of CASHo on sperm concentration

Sperm concentration stabilized after five cycles and remained at a plateau through 25 cycles in both semen stored for less than 5 hours and semen stored for 24 hours (Fig 1). For samples analyzed within 5 hours of collection, ~~For samples analyzed within 5 hours of collection,~~ sperm concentration did not change compared with 0 cycles compared with 0 cycles (*P* = 0.162, 0.155, 0.149, 0.123, and 0.140 for 5, 10, 15, 20 and 25 cycles, respectively). The mean differences compared with 0 and 5 cycles were 30e^8^ and 0.08e^8^ spz/ml, respectively. Given the low concentration values and absence of significant variation, five cycles were considered sufficient for homogenization of semen stored for less than 5 hours.

**Fig. 1. Sperm concentration in semen samples stored for less than five hours or after 24 hours of refrigeration.** Sperm density was counted with UPSPERM after different numbers of mixing cycles in CASHo. Each dot represents the average of five semen samples; vertical lines indicate the standard deviation. ~~Samples from five boars were each split into six aliquots, which were subjected to 0, 5, 10, 15, 20, or 25 cycles.~~ Asterisks indicate differences versus 0 cycles (p < 0.05, one-way ANOVA followed by Dunnett’s test).

For samples stored for 24 hours, sperm concentration reached a plateau after 5 to 10 cycles. Significant differences were detected compared with 0 cycles (*P* = 0.019, 0.002, 0.003, 0.001 and 0.002 for 5, 10, 15, 20 and 25 cycles, respectively). Comparisons with 5 cycles showed no significant differences (P = 0.959, 0.978, 0.906, 0.933 for 10, 15, 20 and 25 cycles, respectively). The mean differences compared with 0 and 5 cycles were 4.3e^8^ and 0.9e^8^ spz/ml, respectively. Thus, five cycles were also considered sufficient for homogenization of semen stored for 24 hours. These findings indicate that CASHo achieves effective homogenization with minimal cycles, making the procedure suitable for application in semen dose production.

## Effects of 5-HT and homogenization cycle on sperm motility kinematics

Once CASHo was validated and the conditions for objective homogenization were established (five cycles for 4.9 ± 2.9 s with an inclination angle of 180^o^), this protocol was applied to investigate the effects of 5-HT on sperm motility kinematics. After the semen samples were homogenized for five cycles, 5-HT was added, and samples were subsequently homogenized for 5, 10, 15, 20, or 25 cycles to mix the additive. The treatment with five cycles was served as the reference condition; whereas the additional cycles (10–25) represented over-homogenization ~~intended to evaluate whether excessive mechanical mixing during incubation affects sperm motility or the response to 5-HT~~.

Two statistical analysis were employed to evaluate (a) the effects of 5-HT and homogenization cycles on the average values of motility parameters ~~in the full dataset~~, and (b) the effects within specific kinematic subpopulations. ~~In both approaches, boar identity was included in the model to account for biological variability.~~

Comparisons were first conducted between the 5-HT group and the vehicle (Vh) group in semen samples subjected to five cycles, followed by analyses of the effects of 5-HT across increasing cycle numbers, using five cycles as the reference. The 95% confidence intervals (CI) (Fixed effects / Interaction) for all analysis are provided in S2 Table. The 95% confidence intervals (CI) for fixed effects and interactions are provided in S2 Table. To facilitate interpretation, emphasis was placed on the magnitude and precision of these effects rather than on statistical significance alone, allowing the assessment of effect sizes and their biological relevance.

### ~~Average of motility parameters~~

### ~~Average values of motility parameters at five cycles~~

Exposure to 5-HT did not significantly affect the percentage of total motility (*p* = 0.681) or progressive motility (*p* = 0.359). Similarly, average values of most kinematic parameters were unaffected (*p* = 0.310, 0.421, 0.686, 0.650, 0.213, and 0.659 for VCL, VAP, VSL, LIN, STR, WOB, and ALH, respectively). However, a significant effect was showed for BeatCross (*p* = 1.89e-05).

### ~~Average values of motility parameters in conditions of over-homogenization~~

Sperm exposed to 5-HT exhibited reduced total and progressive motility at 25 cycles compared with control at same number of cycles (Fig 2). According to the ANOVA results, neither the effect of cycles nor the interaction between treatment (5-HT vs. vehicle) and cycles was significant for total or progressive motility. However, significant effects of 5-HT, cycles, and their interaction were observed for all motility parameters except VCL, LIN, and STR (Fig 3); Individual p-values for the effects of treatment, cycles, and their interaction (cycles × treatment) are provided in S2 Table.

**Fig. 2. Effects of 5-HT and homogenization cycle on sperm motility** (A) Sperm total and progressive motility. The effect of 5-HT at five cycles was compared using Student’s t-test. The effects of 5-HT and number of cycles under over-homogenization conditions were analyzed using a mixed-effects model, with five cycles as the reference level. Data are mean +/- standard error (N = 5). (B) Heatmap showing *p-values* for the effects of 5-HT, homoginzation cycle and their interaction ~~global effects. (B) Heatmap showing p-values for the global effects. The color of each square indicates whether the p-value for treatment, number of cycles, or their interaction falls below or above the significance threshold of 0.05. Numbers inside each square represent the exact~~ *~~p-value.~~*

**Fig. 3. The exposure to 5-HT and distinct number of cycles affected the values of motility parameters.** (A) Interaction plots showing average of kinematic parameters. The effect of 5-HT at five cycles was compared using Student’s t-test. The effects of 5-HT and number of cycles under over-homogenization conditions were analyzed using a mixed-effects model, with five cycles as the reference level. Each dot represents the mean of five semen samples; vertical lines indicate the standard error. (B) Heatmap showing p-values for the global effects. The color of each square indicates whether the p-value for treatment, number of cycles, or their interaction falls below or above the significance threshold of 0.05. Numbers inside each square represent the exact *p-value.*

## Effects of 5-HT and homogenization cycle on sperm kinematic subpopulations

A dataset comprising 18,644 sperm was generated, with motility parameters from each spermatozoon used as input for subpopulation identification. Principal component analysis (PCA) revealed that PC1 and PC2 together explained 78.71% of the variance (Table 2). Individual kinematic parameters displayed distinct associations with specific principal components (Table 2, Fig 4A). The scores for PC1 and PC2 were subsequently used as input for hierarchical clustering. Based on the inertia gain criterion, three clusters were identified, corresponding to distinct kinematic subpopulations (Fig 4B). These subpopulations are illustrated on the principal component map (Fig 4C).

| **Table 2. Detail of Principal components analysis realized on motility parameters obtained from the computer assisted sperm analysis system.** | | |
| --- | --- | --- |
|  | **PC1** | **PC2** |
| **Variance explained (eigenvalue)** | 43.37 (3.47) | 35.34 (2.83) |
| **Eigenvectors *** |  |  |
| **VCL** | 4.24 | 26.78 |
| **VAP** | 17.10 | 12.39 |
| **VSL** | 25.54 | 0.25 |
| **LIN** | 15.91 | 14.56 |
| **STR** | 6.21 | 18.13 |
| **WOB** | 14.60 | 2.94 |
| **BeatCross** | 6.81 | 3.27 |
| **ALH** | 9.59 | 21.66 |
| * Eigenvectors represent a degree of association with each one of motility descriptors.  VCL, curvilinear velocity; VAP, average path velocity; VSL, straight line velocity; LIN, linearity; STR, straightness; WOB, wobble; BeatCross, beat cross frequency; ALH, lateral head displacement. | | |

**Fig 4. Principal component analysis (PCA) was applied to reduce the dimensionality of the dataset, and hierarchical clustering was then used to identify kinematic subpopulations.** Data from 18,644 sperm, each characterized by eight motility parameters, served as input for the principal component analysis (dimensionality reduction step). (A) Correlations between motility parameters and Principal Components 1 and 2. Motility parameters are represented as vectors (arrows); therefore, the direction and length of each arrow indicate the sign and magnitude of the correlation. Data from the principal components were then used as input for the hierarchical clustering algorithm. (B) The number of clusters (branches into color rectangles) was determined based on the dendrogram and the inertia gain (inset bar plot). Inertia refers to variance, with total inertia (total variance) decomposed into between- and within-group components. The within-cluster inertia gain characterizes cluster homogeneity. Labels at the base of the dendrogram correspond to individual sperm IDs. (C) Scatterplot of the first two principal components showing clusters identified in (B), color-coded according to cluster assignment.

The number of sperm in Clusters 1, 2 and 3 was 7,840, 6,611, and 4,193, respectively. The proportions of sperm in the distinct clusters, treatment, and cycles are presented in Fig 5. A Pearson’s chi-square test was conducted to evaluate the independence between 5-HT treatment and sperm subpopulation membership. The test indicated a significant deviation from independence (*p* = 0.031) (Fig 5A). Examination of standardized residuals identified Cluster 3 as the primary contributor to the observed effect. Under 5-HT treatment, the proportion of sperm assigned to Cluster 3 was significantly higher than expected (z = 2.62), while the vehicle group exhibited a significantly lower-than-expected proportion in the same cluster (z = –2.62). The standardized residuals for Clusters 1 and 2 were below the threshold for statistical relevance (|z| < 2). These results support the presence of a treatment-dependent redistribution of sperm among kinematic clusters, with 5-HT stimulation increasing the representation of Cluster 3.

A hierarchical mosaic plot was constructed to examine the joint distribution of sperm across kinematic clusters, treatment, and cycles (Fig 5B). The plot reflects the structure of a log-linear model assuming complete independence among the three categorical variables. Visual inspection of the shaded mosaic revealed deviations from expected frequencies under the independence model, particularly in the Cluster 3 × Treatment × Cycle combinations. Overrepresentation of 5-HT-treated sperm in Cluster 3 was detected at 10 cycles, whereas underrepresentation of vehicle-treated sperm in Cluster 3 was detected at 20 and 25 cycles. The residual patterns did not indicate substantial deviations in Clusters 1 or 2. These results suggest that the effect of treatment on sperm subpopulation distribution is not uniform across the cycles; rather, 5-HT stimulation appears to preferentially affect the dynamics of the subpopulation defined by Cluster 3 in a cycle-dependent manner.

**Fig. 5. Proportion of sperm within clusters in relation to the effects of 5-HT and homogenization cycle.** (A) Mosaic plot showing the proportion of sperm classified into each cluster (1, 2, and 3) for the control (Vh) and 5-HT treatment groups. The height of each tile represents the relative frequency of each treatment–cluster combination, while the width reflects the proportion of sperm assigned to each cluster. (C) Mosaic plot showing distribution of sperm across treatments and homogenization cycles (5, 10, 15, 20, and 25) within each cluster. The blue tiles indicate more sperm than expected under independence (positive residuals), red tiles indicate fewer sperm than expected (negative residuals), and grey tiles indicate values close to the expected distribution. The area of each tile represents the joint frequency of sperm within a given treatment–cycle–cluster combination. The color scale is shown in the lookup table to the right of the mosaic [18].

### Cluster-specific average values of motility parameters at five cycles

Exposure to 5-HT differentially and significantly affected the average values of motility parameters across clusters. Based on the patterns of velocity and linearity values, the clusters were named as follows: Cluster 1, “Low velocity–intermediate linearity”; Cluster 2, “High velocity–low linearity”; and Cluster 3, “Intermediate velocity–high linearity.” In Cluster 1, significant effects were detected for VCL, VAP, VSL, WOB, and ALH (p = 1.89e-15, 3.87e-13, 1.39e-4, 0.048, and 1.09e-15, respectively), whereas LIN and BeatCross were not significantly affected (*p* = 0.935 and 0.419, respectively). In Cluster 2, 5-HT significantly influenced all motility parameters (*p* = 1.95e-4, 1.41e-12, 0.027, 4.98e-5, 3.39e-4, 5.23e-9, and 1.45e-6 for VCL, VAP, LIN, STR, WOB, BeatCross, and ALH, respectively), with the exception of VSL (*p* = 0.743). In Cluster 3, most parameters were not significantly affected (*p* = 0.548, 0.112, 0.089, 0.393, 0.069, and 0.412 for VCL, VAP, VSL, STR, WOB, and ALH, respectively); however, significant effects were detected for LIN and BeatCross (*p* = 0.048 and 6.4e-4, respectively).

### Cluster-specific average values of motility parameters in conditions of over-homogenization

Motility parameter values of sperm treated with 5-HT were affected by the number of cycles, with significant interaction effects between cycles and 5-HT observed under over-homogenization conditions in all clusters (Fig 6). In Cluster 3, no significant effects of 5-HT were detected at 5, 20, and 25 cycles, although WOB and BeatCross remained sensitive to the effects of cycles and to the cycles × 5-HT interaction. Individual p-values for the effects of treatment, cycles, and their interaction (cycles × treatment) are provided in S2 Table.

**Fig. 6.** Effects of 5-HT and homogenization cycles on sperm motility kinematic for each sub-population cluster. (A, C and E) Interaction plots showing average of kinematic parameters of sperm classified into each cluster (1, 2, and 3) for the control (Vh) and 5-HT treatment groups. ~~The effect of 5-HT at five cycles was compared using Student’s t-test.~~ The effects of 5-HT and number of cycles under over-homogenization conditions were analyzed using a mixed-effects model, with five cycles as the reference level. Data are mean +/- standard error. (B, D and F) Heatmap showing *p-values* for the global effects into each cluster (1, 2, or 3). ~~The color of each square indicates whether the~~ *~~p-value~~* ~~for treatment, number of cycles, or their interaction falls below or above the significance threshold of 0.05. Numbers inside each square represent the exact~~ *~~p-value~~*~~.~~

# Discussion

A device capable of objectively homogenizing semen samples was designed and constructed, with control over angle, velocity and number of cycles. The hardware and firmware of CASHo provide precision and repeatability, ensuring semen samples reach a state in which no further changes in sperm density occur. CASHo represents a valuable alternative to commercial or open-hardware devices not specifically intended for semen homogenization. This assessment is based on technical information provided by manufacturers, as no direct experimental comparisons with commercial devices were performed in this study.

**Capabilities and limitations of CASHo**

The designed CASHo possesses various advangates including (1) a highly accurate angle of rotation as a stepper motor was used. . (2) The coefficient of variation in timing between cycles is very low, confirming high repeatability. (3) The angle and duration of rotation are highly precise. These enables objective and reproducible studies on semen homogenization by allowing precise control of rotation speed, angle, and number of cycles. Use of CASHo in experimental settings highlights the importance of identifying the point at which homogenization no longer alters sperm density, allowing the procedure to be stopped once an objective and repeatable state is reached.

Prospective:

- CASHo is an affordable homogenizer designed for boar semen in its current version but can be readily adapted for semen from other species.
- The 50 mL tube holder can be replaced with holders of other capacities (e.g., 2 mL or 15 mL).
- The extruded aluminum base can be substituted with other readily available materials, such as rectangular steel tubing or wood, and secured by welding or screwing.
- CASHo can be upgraded by modifying the firmware and incorporating a screen and potentiometers to regulate the number of routines, cycles, rotation angle, or speed.

## Limitations of existing commercial homogenization devices

In previous studies, the issue of semen homogenization has been addressed experimentally [20–24]; however, none of these studies provided an operational definition or specified a standardized protocol to homogenize semen samples. In some reports, homogenization was performed using equipment not designed for handling sperm samples, or equipment specifically designed for semen samples from a particular species [22,23]. This variation complicates replication and prevents the establishment of standardized homogenization protocols. Moreover, existing commercial equipment tested in prior reports [20-24] cannot be easily or adequately adapted to the requirement of different species.

Although no direct comparison with commercial homogenizers was conducted, our discussion of existing devices and their reported effects is based on previously published studies. Therefore, claims regarding their limitations should be interpreted in this context, and future work should include direct comparative testing to validate these observations experimentally. In the present study, the displacement velocity and rotation angle were determined empirically based on hand-operated trials, before programming CASHo. The effect of manual versus mechanical homogenization on sperm motility was addressed early on; however, the study was conducted under conditions that are difficult to compare. Sperm density, as well as total and progressive motility evaluated by CASA, were reported to be higher in diluted boar semen samples mixed with a vortex mechanical mixer (80% speed) compared to manual mixing (turning the tube approximately five times for 2–3 seconds). Homogeneity of the suspension in that study was assessed visually [20]. The homogenization-like process has been described without specifying the approximate degrees of inclination: agitated by gently rocking each tube five times once daily' [3]. When semen samples are processed in this manner, no effects on sperm motility kinematics were detected, but manual rotation at 180° every 12 hours or five automatic rotations of 360° per hour using a Kobimove Mini (Cobiporc, Saint Gilles, France) decrease boar sperm motility [24]. Similarly, equine sperm subjected to a rotation frequency of 5 revolutions per minute (rpm) and 36 rpm using a roller bench (model RM5, Minitüb GmbH) for four consecutive days showed reduced total motility [25].

In the present study, the serotonin experiment was conceived as a functional demonstration of CASHo’s utility. CASHo was used to assess the effect of homogenization cycles on the motility parameters of sperm exposed to 5-HT. Two complementary analytical approaches were employed including evaluation of overall average values of sperm motility kinematics, and assessment within defined kinematic subpopulations.

## Average values of motility parameters versus subpopulation analysis

The low values of total and progressive motility measured by CASA in this study contrast with the threshold values established by CEIEEP. However, CASA systems generally report lower sperm motility compared to subjective evaluation by technicians [26]. With the CASA plugin used in this study, the main limiting factor reducing the number of motile sperm was the threshold set for the minimum number of tracks required to be classified as motile.

The analysis of average data showed no effects of 5-HT on sperm motility when semen samples were well-homogenized with five cycles. However, the clustering analysis allowed the identification of sperm motility kinematic subpopulations that responded differentially to 5-HT. Moreover, the motile behavior of these sperm subpopulations was differentially affected by 5-HT under over-homogenization conditions. The proportion of sperm in subpopulation 2 was over-represented at five and 25 cycles but under-represented at 10 cycles, indicating a redistribution of sperm among subpopulations as a function of the homogenization conditions. Not all studies using CASA systems identify motility kinematics subpopulations; instead, many report only average values. It has been reported that sperm express proteins related to serotonergic communication, including receptors, transporters, and metabolizing enzymes [11]. In that study, human sperm were exposed to 5-HT under non-capacitating conditions, and motility was evaluated using CASA, but only average motility values were reported. Exposure to 5-HT increased sperm velocities (VCL, VAP, and VSL) [11]. Other studies have reported average motility values for sperm exposed to agonists or antagonists of 5-HT receptors under capacitating conditions [12,13].

In the present work, values of VCL and VAP were lower after exposure to 5-HT at five cycles in Clusters 1 and 2, but no changes were detected in Cluster 3 or when the average values in the full dataset were analyzed. The fact that changes in values of motility parameters were evident at the subpopulational level indicates that relevant information is lost when only average values are considered in the full dataset. Previously, changes in the structure of kinematic subpopulations were detected in boar sperm exposed to ketanserin (a 5-HT₂ receptor inhibitor) under non-capacitating conditions [14]. In that study, the subpopulation with the highest velocity values showed a significant increase in these parameters after exposure to ketanserin (Supplementary data in Rodríguez-Martínez, 2023), which support our results.

Sperm velocity is of biological importance, and a key determinant for fertility success [27]. In this study, the effects of 5-HT on motility parameters varied among clusters. In the cluster with the highest VCL values at five cycles, all motility parameters except VSL showed significant differences. In that cluster, as the number of cycles increased (over-homogenization conditions), interactions with 5-HT effects became evident. These findings suggest that subpopulations have differential responses to 5-HT exposure, manifested as changes in kinematics. The molecular pathways and components underlying these 5-HT-induced changes in kinematics remain unknown. However, the existence of differentially responsive kinematic subpopulations may be linked to sperm development and maturation in the testis and epididymis, respectively [28], as well as to the factors involved and their level of expression.

## 5-HT and hyperactivated sperm motility

Capacitation is a key step in the fertilization process [29]; thus, extenders for long-term storage must prevent capacitation while maintaining motility. One event in the capacitation process is the acquisition of hyperactivated motility. Although serotonergic communication has shown to stimulate sperm hyperactivation [12,13], the 5-HT signaling for hyperactivation were not subjected to the present study. It has been shown that 5-HT stimulate Ca^2+^ signaling, in vitro, to triggers sperm motility hyperactivation [30]. Semen extenders may contain chelating agents, such as EDTA, which have high affinity for Ca^2+^ [31,32]; therefore, 5-HT-related hyperactivated motility is unlikely under the conditions of this study. Another factor required for hyperactivation is prolonged incubation [12,13,30], which is not included. Finally, the characteristic motility patterns described for hyperactivated sperm [12,30,33] were absent.

## Conclusions and future directions

CASHo is a reliable open-hardware device for homogenization of semen samples.

An objective definition of homogenization of boar semen samples can be achieved using CASHo.

Exposure of sperm to 5-HT at five cycles differentially modified motility parameters among subpopulations, indicating that sensitivity of sperm to 5-HT varies across clusters.

5-HT reduced motility parameter values in the subpopulation with highest VCL.

Over-homogenization introduced bias into kinematic results when sperm were exposed to 5-HT.

The effects of 5-HT and over-homogenization on sperm quality parameters other than motility remain unknown. Future research should investigate whether these factors influence sperm viability, membrane integrity, acrosome status, mitochondrial function, or DNA fragmentation, as their absence represents a limitation of the present study.

Because the number of cycles required to reach objective homogenization may vary across species, the threshold at which over-homogenization occurs is also likely to differ. Exploring these additional endpoints will help determine whether the effects detected on motility extend to other aspects of sperm physiology. Furthermore, studies across different species and extender formulations could clarify whether the impact of homogenization cycles and serotonergic signalling is generalizable or context-dependent.

# Acknowledgments

The authors thank Martin Lavalle for his assistance in preparing isometric and technical draws of the STL models.

**Ethics statement**

Semen doses intended for artificial insemination were donated by the CEIEEP. Thus, this work not involved animals.

**Author Contributions**

**Nancy Juarez**: Methodology, Investigation, Writing – review & editing. **Cindy Rivas**: Methodology, Investigation, Writing – review & editing. **Oscar Gutiérrez**: Methodology. **María Ayala**: Investigation, formal analysis, Writing – review & editing. **Andrés Aragón**: Conceptualization, Funding Acquisition, Supervision, Writing-Review & Editing.

# References

1.	Rivas C, Ayala M, Aragon A. Effect of various pH levels on the sperm kinematic parameters of boars. South Afr J Anim Sci. 2022;52: 693–704. doi:http://dx.doi.org/10.4314/sajas.v52i5.13

2.	Rivas AC, Ayala ME, Aragón MA. A new open-source hardware device to measure vertical sperm motility and concentration. Cytometry A. 2021. doi:https://doi.org/10.1002/cyto.a.24343

3.	Balogun KB, Stewart KR. Effects of air exposure and agitation on quality of stored boar semen samples. Reprod Domest Anim. 2021;56: 1200–1208. doi:10.1111/rda.13975

4.	Vyt P, Maes D, Sys S, Rijsselaere T, Van Soom A. Air Contact Influences the pH of Extended Porcine Semen. Reprod Domest Anim. 2007;42: 218–220. doi:10.1111/j.1439-0531.2006.00733.x.

5.	Schulze M, Bortfeldt R, Schäfer J, Jung M, Fuchs-Kittowski F. Effect of vibration emissions during shipping of artificial insemination doses on boar semen quality. Anim Reprod Sci. 2018;192: 328–334. doi:10.1016/j.anireprosci.2018.03.035

6.	Tamanini MSC, dos Santos G, Leal LA, Wolf LM, Schulze M, Christ TS, et al. Impact of agitation time of boar semen doses on sperm traits in short- and long-term extenders. Anim Reprod Sci. 2022;247: 107159. doi:10.1016/j.anireprosci.2022.107159

7.	Paschoal AFL, Luther A-M, Jakop U, Schulze M, Bortolozzo FP, Waberski D. Factors influencing the response of spermatozoa to agitation stress: Implications for transport of extended boar semen. Theriogenology. 2021;175: 54–60. doi:10.1016/j.theriogenology.2021.08.033

8.	Wang S, Zeng X, Liu S, Hoque SAM, Min L, Ding N, et al. Vibration Emissions Reduce Boar Sperm Quality via Disrupting Its Metabolism. Biology. 2024;13: 370. doi:10.3390/biology13060370

9.	WHO. WHO laboratory manual for the Examination and processing of human semen. 5th ed. Switzerland: World Healt Organization; 2010.

10.	Dhankani KC, Pearce JM. Open source laboratory sample rotator mixer and shaker. HardwareX. 2017;1: 1–12. doi:10.1016/j.ohx.2016.07.001

11.	Jiménez-Trejo F, Coronado-Mares I, Boeta M, González-Santoyo I, Vigueras-Villaseñor R, Arriaga-Canon C, et al. Identification of serotoninergic system components in stallion sperm. Histol Histopathol. 2018;33: 951–958. doi:10.14670/HH-11-989

12.	Fujinoki M. Serotonin-enhanced hyperactivation of hamster sperm. Reproduction. 2011;142: 255–266. doi:10.1530/REP-11-0074

13.	Sakamoto C, Fujinoki M, Kitazawa M, Obayashi S. Serotonergic signals enhanced hamster sperm hyperactivation. J Reprod Dev. 2021;67: 241–250. doi:10.1262/jrd.2020-108

14.	Rodríguez-Martínez EA, Rivas CU, Ayala ME, Blanco-Rodríguez R, Juarez N, Hernandez-Vargas EA, et al. A new computational approach, based on images trajectories, to identify the subjacent heterogeneity of sperm to the effects of ketanserin. Cytometry A. 2023;103: 655–663. doi:10.1002/cyto.a.24732

15.	Dziekońska A, Fraser L, Majewska A, Lecewicz M, Zasiadczyk Ł, Kordan W. Effect of commercial long-term extenders on metabolic activity and membrane integrity of boar spermatozoa stored at 17 degrees C. Pol J Vet Sci. 2013;16: 517–525. doi:10.2478/pjvs-2013-0072

16.	Edelstein AD, Tsuchida MA, Amodaj N, Pinkard H, Vale RD, Stuurman N. Advanced methods of microscope control using μManager software. J Biol Methods. 2014;1: 10. doi:10.14440/jbm.2014.36

17.	Rasband WS. ImageJ, US National Institutes of Health. Bethesda, Maryland, USA; 1997. Available: https://imagej.nih.gov/ij/

18.	Friendly M. Mosaic Displays for Multi-Way Contingency Tables. J Am Stat Assoc. 1994;89: 190–200. doi:10.2307/2291215

19.	R Core Team. R: A Language and Environment for Statistical Computing. Vienna, Austria; 2017. Available: https://www.R-project.org/

20.	Menegat MB, Mellagi APG, Bortolin RC, Menezes TA, Vargas AR, Bernardi ML, et al. Sperm quality and oxidative status as affected by homogenization of liquid-stored boar semen diluted in short- and long-term extenders. Anim Reprod Sci. 2017;179: 67–79. doi:10.1016/j.anireprosci.2017.02.003

21.	Schulze M, Rüdiger K, Waberski D. Rotation of Boar Semen Doses During Storage Affects Sperm Quality. Reprod Domest Anim Zuchthyg. 2015;50: 684–687. doi:10.1111/rda.12532

22.	Bakst MR, Cecil HC. Research Note: Effects of Agitation and Temperature Changes on Turkey Sperm Viability After Twenty-Four Hour Storage. Poult Sci. 1992;71: 395–397. doi:10.3382/ps.0710395

23.	Rodríguez-Gil JE, Rigau T. Effects of slight agitation on the quality of refrigerated boar sperm. Anim Reprod Sci. 1995;39: 141–146. doi:10.1016/0378-4320(95)01390-L

24.	Pommerehn L, Menegat MB, Mellagi APG, Rodríguez G, Bernardi M, Wentz I, et al. INFLUÊNCIA DA FREQUÊNCIA DE AGITAÇÃO NA QUALIDADE DE DOSES DE SÊMEN SUÍNO EM DIFERENTES DILUENTES DE CONSERVAÇÃO. Suinocultura. Campinas, SP; 2017. Available: https://pt.engormix.com/suinocultura/artigos/influencia-frequencia-agitacao-qualidade-t40030.htm

25.	Dierberger H, Pieper L, Jung M, Schulze M. Rotation of liquid-preserved artificial insemination doses on roller benches affects sperm quality during storage in stallions. Reprod Domest Anim. 2023;58: 1413–1419. doi:10.1111/rda.14456

26.	Tomlinson MJ, Pooley K, Simpson T, Newton T, Hopkisson J, Jayaprakasan K, et al. Validation of a novel computer-assisted sperm analysis (CASA) system using multitarget-tracking algorithms. Fertil Steril. 2010;93: 1911–1920. doi:10.1016/j.fertnstert.2008.12.064

27.	Donnelly ET, Lewis SE, McNally JA, Thompson W. In vitro fertilization and pregnancy rates: the influence of sperm motility and morphology on IVF outcome. Fertil Steril. 1998;70: 305–314. doi:10.1016/s0015-0282(98)00146-0

28.	Martínez-Pastor F. What is the importance of sperm subpopulations? Anim Reprod Sci. 2022; 106844. doi:10.1016/j.anireprosci.2021.106844

29.	Schmid S, Henning H, Petrunkina AM, Weitze KF, Waberski D. Response to capacitating stimuli indicates extender-related differences in boar sperm function. J Anim Sci. 2013;91: 5018–5025. doi:10.2527/jas.2013-6287

30.	Harayama H. Flagellar hyperactivation of bull and boar spermatozoa. Reprod Med Biol. 2018;17: 442–448. doi:10.1002/rmb2.12227

31.	Johnson LA, Weitze KF, Fiser P, Maxwell WM. Storage of boar semen. Anim Reprod Sci. 2000;62: 143–172. doi:10.1016/s0378-4320(00)00157-3

32.	Gadea J. Review: semen extenders used in the artificial inseminarion of swine. Span J Agric Res. 2003;1: 17–27. doi:10.5424/sjar/2003012-17

33.	Suarez SS, Ho HC. Hyperactivated motility in sperm. Reprod Domest Anim. 2003;38: 119–124. doi:10.1046/j.1439-0531.2003.00397.x

# Supporting Information

**S1 Table. List of components, cost, source, and material type.**

**S2 Table. Confidence intervals for fixed effects / interaction.**
