# Supplementary material for: Development of an open-hardware semen homogenizer and application to serotonin effects on sperm motility
Source: PLoS One. 2025 Dec 5;20(12):e0338399. doi: 10.1371/journal.pone.0338399 (PMC12680138; doi:10.1371/journal.pone.0338399)
Supplement: Attachment — Submitted filename: Response_to_reviewers_auresp_4.docx [file pone.0338399.s010.docx]

The authors of the manuscript PONE-D-25-30420R3 “Development of an open-hardware semen homogenizer and application to serotonin effects on sperm motility” are grateful with Editor. Each one of the comments and suggestions were addressed, as specified below. We hope this version of our manuscript meet the standards of quality of PLOSONE.

Best regards,

Dr. Andrés Aragón

Very importantly;

1- Please include, a brief description of CASHo and operational information in the supplementary materials, including components that has been provided in the original submission.

**Answer.** Thank you for your recommendation. The supplementary file 1 now include description of CASHo, operational information and components as in the original submission.

2- The most comments from all three reviewers corresponds to the statistical analyses. I have also a big concern. I would appreciate if you double check with following introduction and guide, and revise the MS. For more details, please read the following key reference: Seltman, H. J. 2018. “Two‐Way ANOVA.” In Experimental Design and Analysis, 267–292. Carnegie Mellon University Publication. https:// www.stat.cmu.edu/~hseltman/309/Book/Book.pdf.

The statistical analysis has used two-way or repeated-measure ANOVA; however the method for operation this kind of analysis needs revision.

In the present study, there are two main factors (homogenization cycle and 5-HT). To conduct statistical analysis, very simply apply a two-way ANOVA and investigate the effects of homogenization cycle, 5-HT, and their interactions (homogenization cycle x 5-HT). THIS HAS BEEN DONE. However, when interaction effects of 5-HT x cycle is significant for a parameter (such as VCL, VSL, … in your case), then it is enough to conduct one-way ANOVA and compare each parameter between 5-HT and vehicle AT EACH CYCLE (We already know that 5 cycle is well enough for sperm homogenization). To better get familiar with this kind of analysis; I have included two articles form our works that provide step-by-step stat procedures. This is not to cite these works as references, I am not going to receive bias citation. It is just for your kind considerations to revise the MS correctly, and present the findings in a correct form.

(1) https://doi.org/10.1371/journal.pone.0243569

(2) https://doi.org/10.1002/jez.2918

**Answer.** We are grateful for your comments and for the references you provided. We reviewed them carefully and revised our statistical procedure accordingly. In the studies you referred to, two explanatory variables with multiple levels were evaluated across different parameters. In our case, we also have two explanatory variables: Treatment with two levels (Vh and 5-HT) and Cycles with five levels (5, 10, 15, 20, and 25). Therefore, for a given motility parameter, when the interaction term in the linear mixed model was significant, we decomposed the analysis by performing one-way ANOVAs to compare the means of the Vh and 5-HT groups at each cycle level.

We incorporated the following text into the Statistics section: “When a significant interaction was detected, the models were further analyzed by performing separate one-way ANOVAs at each cycle level. Conversely, if the interaction was not significant, the main effects of cycles and 5-HT were evaluated directly.”

We also modified the figures containing interaction plots by adding asterisks to indicate differences between the Vh and 5-HT groups at each cycle level. The corresponding figure legends were updated accordingly.

3- There are a large numbers of comments/recommendations to your MS that I have included directly in the file using a track-change. I would appreciate if you kindely and carefully double check them and revise the MS to reach an acceptable version for publication.

**Answer.** Thank you for your helpful comments and recommendations on the manuscript; each of them was carefully addressed. The revised manuscript containing your suggestions has been uploaded as the “Revised Manuscript with Track Changes.” All comments are now marked as “Resolved” in LibreOffice. In some cases, when text was deleted, the comment appears crossed out, but it was nevertheless fully addressed. In the PDF version generated by the PLOS ONE platform, the comments appear with different shading.

4- If you follow comments no. 2; you may find that Supplementary tables 1 and 2 may not be necessary.

**Answer.** Thank you for your comment. We addressed the issues raised in points 1 and 2; therefore, Supplementary Tables 1 and 2 were deleted. The S1 File now contains the description, operational information, and components of CASHo. In accordance with point 2, S2 Table was also removed.

5- To my experience, I could not access the video files you uploaded on OSF. I would suggest to support your submission with adding the videos into supplementary materials of your MS.

**Answer.** Thank you for your kind comment. You are right—the videos cannot be viewed online on OSF. I have reported this issue to the OSF administrator, and their engineers are currently working to resolve it. In the meantime, I have added the following note to the “Videos” section of the project Wiki: “If the videos cannot be viewed online on OSF, please download them.”

We have also added Videos S1 and S2 to the supplementary materials of the manuscript.
